# LncRNA Profiling Reveals That the Deregulation of H19, WT1-AS, TCL6, and LEF1-AS1 Is Associated with Higher-Risk Myelodysplastic Syndrome

**DOI:** 10.3390/cancers12102726

**Published:** 2020-09-23

**Authors:** Katarina Szikszai, Zdenek Krejcik, Jiri Klema, Nikoleta Loudova, Andrea Hrustincova, Monika Belickova, Monika Hruba, Jitka Vesela, Viktor Stranecky, David Kundrat, Pavla Pecherkova, Jaroslav Cermak, Anna Jonasova, Michaela Dostalova Merkerova

**Affiliations:** 1 Institute of Hematology and Blood Transfusion, U Nemocnice 1, 128 20 Prague 2, Czech Republic; katarina.szikszai@uhkt.cz (K.S.); zdenek.krejcik@uhkt.cz (Z.K.); nikoleta.loudova@uhkt.cz (N.L.); andrea.mrhalkova@uhkt.cz (A.H.); monika.belickova@uhkt.cz (M.B.); monika.hruba@uhkt.cz (M.H.); jitka.vesela@uhkt.cz (J.V.); david.kundrat@uhkt.cz (D.K.); pavla.pecherkova@uhkt.cz (P.P.); jaroslav.cermak@uhkt.cz (J.C.); 2 Department of Computer Science, Czech Technical University, 121 35 Prague, Czech Republic; klema@fel.cvut.cz; 3 Faculty of Science, Charles University, 128 00 Prague, Czech Republic; 4 First Faculty of Medicine, Charles University, 121 08 Prague, Czech Republic; vstra@lf1.cuni.cz; 5 General University Hospital, 128 08 Prague, Czech Republic; Anna.Jonasova@vfn.cz

**Keywords:** myelodysplastic syndrome, lncRNA, progression, outcome, pathogenesis, coexression network

## Abstract

**Simple Summary:**

Although lncRNAs have been increasingly recognized as regulators of hematopoiesis, only several studies addressed their role in myelodysplastic syndrome (MDS). By genome-wide profiling, we identified lncRNAs deregulated in various groups of MDS patients. We computationally constructed lncRNA-protein coding gene networks to associate deregulated lncRNAs with cellular processes involved in MDS. We showed that expression of H19, WT1-AS, TCL6, and LEF1-AS1 lncRNAs associate with higher-risk MDS and proposed processes related with these transcripts.

**Abstract:**

Background: myelodysplastic syndrome (MDS) is a hematopoietic stem cell disorder with an incompletely known pathogenesis. Long noncoding RNAs (lncRNAs) play multiple roles in hematopoiesis and represent a new class of biomarkers and therapeutic targets, but information on their roles in MDS is limited. Aims: here, we aimed to characterize lncRNAs deregulated in MDS that may function in disease pathogenesis. In particular, we focused on the identification of lncRNAs that could serve as novel potential biomarkers of adverse outcomes in MDS. Methods: we performed microarray expression profiling of lncRNAs and protein-coding genes (PCGs) in the CD34+ bone marrow cells of MDS patients. Expression profiles were analyzed in relation to different aspects of the disease (i.e., diagnosis, disease subtypes, cytogenetic and mutational aberrations, and risk of progression). LncRNA-PCG networks were constructed to link deregulated lncRNAs with regulatory mechanisms associated with MDS. Results: we found several lncRNAs strongly associated with disease pathogenesis (e.g., H19, WT1-AS, TCL6, LEF1-AS1, EPB41L4A-AS1, PVT1, GAS5, and ZFAS1). Of these, downregulation of LEF1-AS1 and TCL6 and upregulation of H19 and WT1-AS were associated with adverse outcomes in MDS patients. Multivariate analysis revealed that the predominant variables predictive of survival are blast count, H19 level, and TP53 mutation. Coexpression network data suggested that prognosis-related lncRNAs are predominantly related to cell adhesion and differentiation processes (H19 and WT1-AS) and mechanisms such as chromatin modification, cytokine response, and cell proliferation and death (LEF1-AS1 and TCL6). In addition, we observed that transcriptional regulation in the H19/IGF2 region is disrupted in higher-risk MDS, and discordant expression in this locus is associated with worse outcomes. Conclusions: we identified specific lncRNAs contributing to MDS pathogenesis and proposed cellular processes associated with these transcripts. Of the lncRNAs associated with patient prognosis, the level of H19 transcript might serve as a robust marker comparable to the clinical variables currently used for patient stratification.

## 1. Introduction

Myelodysplastic syndrome (MDS) is a heterogeneous group of clonal hematopoietic stem cell (HSC) disorders characterized by bone marrow (BM) dysplasia with ineffective hematopoiesis, peripheral blood cytopenia, and an increased tendency for transformation to acute myeloid leukemia (AML). Pathogenesis of MDS is a multifactorial process in which cytogenetic aberrations, gene mutations, and epigenetic changes are involved. Using the WHO classification criteria [1], MDS patients are classified into various diagnostic subtypes based on the number of affected hematopoietic lineages, the percentage of BM blasts, cytogenetics and the presence of ring sideroblasts. Patient prognosis is evaluated based on the Revised International Prognostic Scoring System (IPSS-R) depending on similar clinicopathological criteria [2]. For higher-risk MDS, treatment with the hypomethylating agent azacitidine (AZA) is currently considered standard therapy, which prolongs patient survival, improves clinical outcomes and quality of life, and delays the disease progression in a proportion of patients [3].

Long noncoding RNAs (lncRNAs) are a group of RNAs that are defined as non-protein-coding transcripts longer than 200 nucleotides. The properties of lncRNAs, such as stability and tissue specificity, make them highly promising diagnostic and prognostic markers as well as interesting therapeutic targets. Although lncRNAs are increasingly recognized as regulators of normal and aberrant hematopoiesis, only several studies have addressed their expression and function in relation to MDS. For example, Liu et al. profiled lncRNA expression and identified several lncRNAs (linc-ARFIP1-4, linc-TAAR9-1, lincC2orf85, linc-RNFT2-1 and linc-RPIA) deregulated in MDS with excess blasts II (MDS-EB2) [4]. Further, Yao et al. established a 4-lncRNA risk score significantly associated with patient survival [5].

Although increasing numbers of deregulated lncRNAs are currently being described in MDS, their functional characterization is still difficult. Transcriptomic data may be used to construct coexpression networks of similarly regulated lncRNAs and protein-coding genes (PCGs), which enables the functional analysis of lncRNAs with unknown functions [4]. This approach uses the “guilt-by-association” strategy working with the principle that genes with related functions tend to have similar expression profiles. Thus, the PCGs in a coexpression module are associated with signaling pathways and Gene Ontology terms, attributing the same functions to the unknown lncRNAs in the network.

In this study, we used a microarray platform to profile lncRNA and PCG expression in parallel in CD34+ BM cells of MDS patients with an emphasis on the identification of lncRNAs with altered levels in various groups of MDS patients. In particular, we aimed to characterize the lncRNAs that could serve as novel potential biomarkers of adverse outcomes in MDS. Moreover, a computational approach for constructing lncRNA-PCG networks was applied to associate these lncRNAs with regulatory mechanisms associated with MDS.

## 2. Patients and Methods

The study included CD34+ BM cells of 183 patient or control samples randomly divided into a discovery cohort (54 MDS patient samples, 14 AML with myelodysplasia-related changes (AML-MRC) patient samples, and 9 healthy control samples) used for the microarray profiling and a testing cohort (79 MDS patient samples, 14 AML-MRC patient samples, and 13 healthy control samples) used for reverse transcription quantitative PCR (RT-qPCR). Informed consent was obtained from all individuals and the study was approved by the Institutional Scientific Board and the IHBT ethic committee on 16/06/2016 (ethic code: EK3/AZV/06/2016) and performed in accordance with the ethical standards of the Declaration of Helsinki. The detailed clinical and laboratory characteristics of both cohorts, including the classification of MDS patients into subgroups, IPSS-R categories, bone marrow features and blood counts, are summarized in Table S1.

Expression profiles were determined using Agilent Human GENCODE Custom lncRNA Expression Microarray Design [6], consisting of probes for 22,001 lncRNA transcripts and 17,535 PCG mRNAs. Bioinformatical analyses were performed with the Bioconductor project in the R statistical environment using the limma package. The raw and normalized data have been deposited in the National Center for Biotechnology Information (NCBI) Gene Expression Omnibus (GEO) database under accession number GSE145733.

RT-qPCR was applied using the TaqMan gene expression system (Thermo Fisher Scientific, Waltham, MA, USA) to measure individual transcript levels (lncRNAs: CHRM3-AS2, EPB41L4A-AS1, H19, LEF1-AS1, PVT1, TCL6, and WT1-AS; PCGs: IGF2, LEF1, WT1, TCL1A, and TCL1B; miRNAs: miR-675 and RNU48 as a reference). For normalization of lncRNA data, several reference genes were tested using the RefFinder tool [7] (Figure S1) and the data were finally normalized to the level of HPRT1.

The TruSight Myeloid Sequencing Panel Kit (Illumina, San Diego, CA, USA) containing 568 amplicons in 54 genes was used for mutational screening. Variants were detected by LoFreq and annotated using Variant Effect Predictor, and their clinical significance was verified in several genomic databases (UCSC, COSMIC, ExAC, and PubMed). The arbitrary cut-off was set at 5% of the variant allele frequency (VAF).

The functional changes related to deregulations in gene expression were assessed using gene set enrichment analysis (GSEA) [8]. The lncRNA-PCG coexpression network analysis was carried out as introduced in [4]. Briefly, we identified differentially expressed lncRNAs and PCGs (FDR < 0.05) and constructed a correlation matrix for these transcripts. Then, non-negative matrix factorization (NMF) was used to extract modules from the correlation matrix and each module was functionally annotated by mapping of PCGs to GO terms. The representative cores of the individual modules were visually plotted (for only 4 lncRNAs and 13 PCGs with the highest module membership).

All other statistical analyses were performed using GraphPad Prism 7 (GraphPad Software, La Jolla, CA, USA) and SPSS software (IBM, Armonk, NY, USA).

The detailed version of the Methods is included in the manuscript as a supplementary file (Supplementary Methods).

## 3. Results

### 3.1. MDS-Specific Transcriptome

The gene expression profiles of PCGs and lncRNAs were examined in the CD34+ BM cells of MDS patients. The discovery cohort used for the microarray profiling included 54 patients with MDS, 14 patients with AML-MRC, and 9 healthy donors (Table S1). In summary, we detected a signal of 29,604 transcript probes (out of 61,538 probes spotted on the array). After merging sequence duplicates, probes for 12,444 PCGs and 14,518 lncRNAs were detected. To compare the effect of PCGs and lncRNAs on the disease, we analyzed the data for both categories of transcripts separately.

First, we evaluated gene expression changes between MDS patients and healthy individuals. In MDS, we found 32 lncRNAs (28 upregulated/4 downregulated) and 87 PCGs (83 upregulated/4 downregulated) significantly deregulated compared to those in controls (|logFC| > 1, FDR < 0.05) (Table S2). Of the functionally described lncRNAs, we detected the upregulation of H19, EMCN-IT1, WT1-AS, MEG8, and PVT1 and the downregulation of ST6GAL2-IT1 and U3. Second, the expression of 11 lncRNAs (all 11 downregulated, e.g., VPS9D1-AS1, PVT1, and CXADRP3) and 161 PCGs (2 upregulated/159 downregulated) was significantly changed in AML-MRC compared to that in MDS (Table S3).

**Figure 1:**
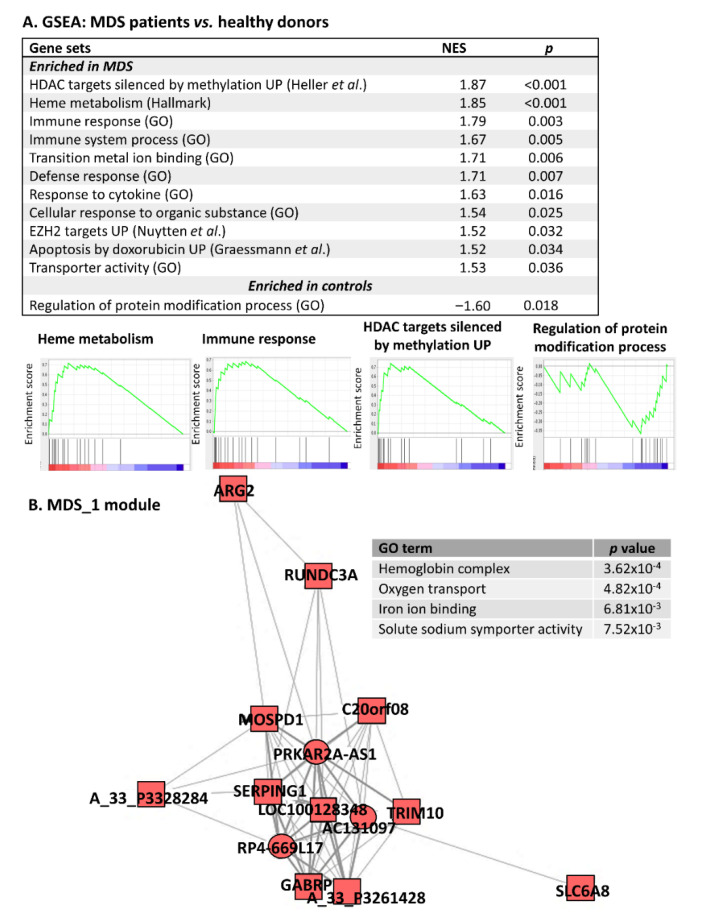
Pathway analysis of genes deregulated between MDS patients and healthy donors. (**A**) Gene set enrichment analysis (GSEA) of differentially expressed PCGs and four enrichment plots for selected enriched gene sets. NES - normalized enrichment score. References: Heller et al. [9], Nuytten et al. [10], and Graesmann et al. [11]. (**B**) MDS_1 module from the lncRNA-PCG coexpression network. The Gene Ontology (GO) terms significantly associated with these modules are listed in the corresponding table. Square-PCG, circle-lncRNA, red-upregulated in MDS.

Gene set enrichment analysis (GSEA) identified 12 gene sets enriched (Figure 1A) in MDS CD34+ cells compared to healthy cells. Overall, the enriched processes were mainly related to four mechanisms: (i) hemoglobin complex and oxygen transport, (ii) immune response, (iii) epigenetic modifications, and (iv) regulation of gene expression. To assess the functions of the deregulated lncRNAs, we built the lncRNA-PCG coexpression network. Within this network, we defined individual modules (assigned as MDS modules) and recognized those associated with the abovementioned processes enriched in MDS. In the cores of these modules, we identified lncRNAs whose expression was substantially correlated with the expression of core PCG nodes and might therefore be linked to deregulated processes. Table 1 summarizes enriched processes and core PCGs and lncRNAs within selected modules and Figure 1B shows an illustrative module of the lncRNA-PCG coexpression network (i.e., MDS_1 module).

Two modules (MDS_1 and MDS_2) were assigned to hemoglobin and oxygen transport. Both of them included mostly upregulated genes (Table 1), suggesting that the ineffective erythropoiesis found in MDS can result in the increased transcription of multiple factors associated with oxygen transport. Based on significant coexpression, the following lncRNAs were recognized as potentially associated with alterations in oxygen transport machinery: e.g., PRKAR2A-AS1, AC131097, and RP4-669L17 in MDS_1 module, and P11-640M9.1, RP11-640M9.1, CTD-2319I12, and RP11-558A11 in MDS_2 module.

Further, we found that two other modules (MDS_3 and MDS_4) were enriched for genes involved mainly in protein metabolism processes (Table 1). MDS_3 module was primarily associated with protein catabolism and the ubiquitin-proteasome pathway and in the core of this module, we identified AC021188 and ZFAS1 lncRNAs. Two other lncRNAs (EPB41L4A-AS1 and CSNK1A1P1) were found in the core of the MDS_4 module that was associated with transcriptional and translational processes. Interestingly, all the genes that were included in the core of MDS_4 module were significantly downregulated, suggesting that the mechanisms of protein expression can be globally suppressed in dysplastic cells.

### 3.2. LncRNA Expression in Relation to the Diagnostic Subtypes of MDS

**Figure 2:**
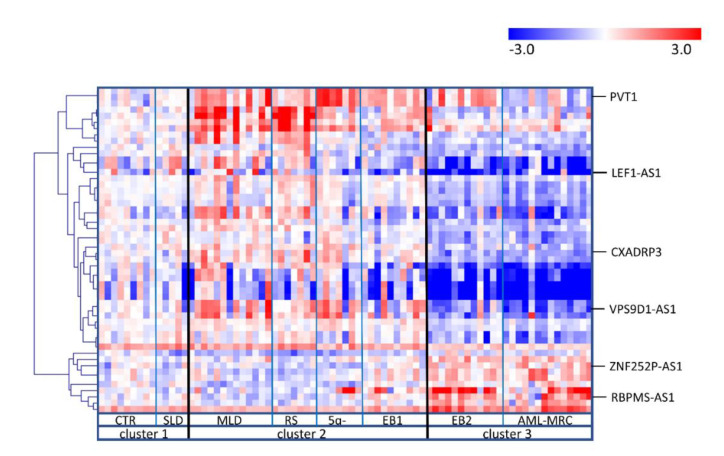
Heatmap of differentially expressed lncRNAs among MDS subtypes. Only the lncRNAs identified as significantly deregulated (FDR < 0.05) in one-way ANOVA were plotted. Samples were divided into eight groups (CTR, MDS-SLD, MDS-MLD, MDS-RS, MDS with del(5q), MDS-EB1, MDS-EB2, AML-MRC), and the analysis defined three clusters of samples with comparable expression profiles. The expression level is calculated as the binary logarithm of fold change (logFC) compared to the mean expression of controls. The heatmap uses a color gradient intensity scale to visually express the logFC values in a range of colors (blue-downregulation, red-upregulation, white-unchanged expression).

Based on the WHO diagnostic criteria [1], eight groups of samples were defined (i.e., CTR, MDS-SLD, MDS-MLD, MDS-RS, MDS with del(5q), MDS-EB1, MDS-EB2, and AML-MRC) and their expression profiles were compared using one-way ANOVA (Figure 2). We identified three clusters of samples. First, MDS-SLD samples had comparable expression profiles to those of healthy controls. Second, the other remaining early disease subtypes (MDS-MLD, MDS-RS, and MDS with del(5q)) surprisingly clustered with samples from the patients with MDS-EB1 disease (i.e., an advanced subtype of MDS). Third, patients with MDS-EB2 showed similar expression to those with AML-MRC. This distribution shows that there are no specific expression profiles for particular disease subtypes but rather that there is a gradual shift in expression from a healthy state to an advanced myelodysplasia. Interestingly, disease progression can be detected at the molecular level at different point (i.e., between MDS-EB1 and MDS-EB2 subtypes) compared to the classical progression scheme created on the basis of clinical variables (i.e., between MDS-EB2 and AML-MRC).

### 3.3. LncRNAs in MDS with Isolated del(5q)

The interstitial deletion of the long arm of chromosome 5, del(5q), is the most common cytogenetic aberration in myelodysplasia, and MDS with isolated del(5q) forms a distinct subtype of this disease [1]. In the discovery cohort, isolated del(5q) was found in 12 MDS/AML-MRC patients, and 20 patients had a normal karyotype. The gene expression profiles of these two groups were compared, and deregulation of 31 lncRNAs (16 upregulated/15 downregulated) and 160 PCGs (106 upregulated/54 downregulated) were identified (|logFC| > 1, FDR < 0.05). The list of deregulated genes is included in Table S4.

Significant deregulation of several hematopoiesis/oncology-related lncRNAs was detected in patients with isolated del(5q), e.g., upregulation of EMCN-IT1, CHRM3-AS2, and PVT1 and downregulation of ZFAS1, EPB41L4A-AS1, and GAS5. The expression levels of CHRM3-AS2, PVT1, and EPB41L4A-AS1 were validated in the testing cohort using RT-qPCR (Figure S2), and the data showed a high level of concordance, indicating the accuracy of the microarray results.

GSEA showed that del(5q) mainly affected genes involved in ribosome formation, translational regulation, STAT5 signaling, and hematopoietic cell lineages including stem/progenitor cells, platelets, and erythrocytes (Figure S3). Further, we constructed a lncRNA-PCG coexpression network, and among the generated modules (assigned as del(5q) modules), we identified those associated with the abovementioned pathways. Based on these modules, we proposed association of several lncRNAs with ribosome formation and translational regulation (del(5q)_1 module, lncRNAs: EPB41L4A-AS1), JAK/STAT cascade (del(5q)_2 module, lncRNAs: NUTM2A-AS1, STARD4-AS1, and RP11-506M13.3) development of blood lineages such as platelet, erythrocyte and myeloid cells (del(5q)_3 module, lncRNAs: BOLA3-AS1, MIR4435-2HG, and ENST433198.2; and del(5q)_4 module, lncRNAs: PVT1, RP11-797H7.5, and RP11-558A11.3), and mitochondria-related processes (del(5q)_5 module, lncRNAs: OIP5-AS1 and POFUT1-006). Table 1 summarizes enriched processes and core PCGs and lncRNAs within these modules.

### 3.4. Association Between lncRNA Expression and Somatic Mutations

Somatic mutations in multiple genes have recently been associated with MDS [12], rapidly becoming the most frequently discussed aberrations in MDS. Here, we investigated the relationship between the presence of somatic mutations and the expression of lncRNAs. Mutational screening was performed in 64 out of 68 patients and in 8 out of 9 controls in the discovery cohort (due to DNA availability). The results showed that 81% of patients bore at least one somatic mutation (VAF > 5%) with 1.9 mutational events per patient on average (range 0–7). The five most frequently mutated genes included SF3B1 (14 patients, 22%), TET2 (10 patients, 16%), TP53 (10 patients, 16%), DNMT3A (9 patients, 14%), and RUNX1 (9 patients, 14%). In contrast, we found no mutations in healthy controls, excluding the presence of clonal hematopoiesis of indeterminate potential (CHIP). The distribution of the detected mutations within the cohort is shown in Figure S4.

The transcriptional effects of somatic mutations were analyzed in the five most frequently mutated genes (SF3B1, TET2, TP53, DNMT3A, and RUNX1). Within differential expression analyses, we searched for the transcripts with differential levels between patients with and without the given mutation. However, the analysis identified only a few transcripts with standard settings (|logFC| > 1, FDR < 0.05). Therefore, we moderately refined the cut-off of fold change values and reanalyzed the data (|logFC| > 0.3, FDR < 0.05). Interestingly, the numbers of differentially expressed genes substantially varied among the mutations tested (SF3B1: 18 lncRNAs and 20 PCGs; TET2: 13 lncRNAs and 5 PCGs; TP53: 8 lncRNAs and no PCGs; DNMT3A: 1 lncRNA and no PCGs; and RUNX1: 106 lncRNAs and 646 PCGs). At the level of individual transcripts related to hematopoiesis/oncology, we observed the downregulation of the ABCB7 PCG in SF3B1-mutated patients, the downregulation of WT1-AS in TET2-mutated patients, and the upregulation of GAS5 lncRNA, the downregulation of LEF1-AS and TCL6 lncRNAs, and the downregulation of LEF1 and RAG1 PCGs in RUNX1-mutated patient. The full lists of significantly deregulated genes in the patients with the five studied mutations are included in Tables S5–S9.

**Figure 3:**
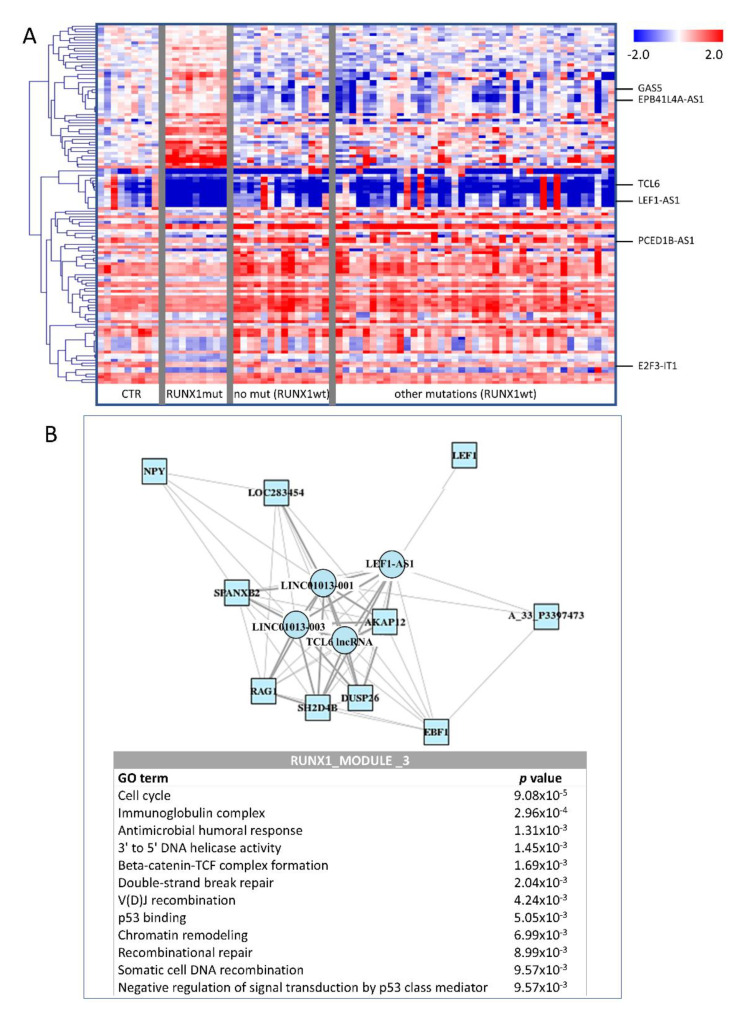
Deregulation of gene expression between the RUNX1-mutated (RUNX1mut) vs. RUNX1-wild type (RUNX1wt) patient samples. (**A**) Heatmap showing the difference in lncRNA expression in MDS/AML-MRC patients stratified according to the presence/absence of the RUNX1 mutation. Only the lncRNAs with significantly changed levels (FDR < 0.05) are plotted. The expression level is calculated as the binary logarithm of fold change (logFC) compared to the mean expression of controls. The heatmap uses a color gradient intensity scale to visually express the logFC values in a range of colors (blue-downregulation, red-upregulation, white-unchanged expression). (**B**) RUNX1_3 module from the lncRNA-PCG coexpression network constructed based on differentially expressed genes. The results of pathway enrichment analysis for this module are included. Square-PCG, circle-lncRNA, red-upregulated in RUNX1mut, blue-downregulated in RUNX1mut.

Because RUNX1-mutated patients displayed the most distinct expression profile, we further focused on this particular gene. A descriptive heatmap (Figure 3A) proved that patients with RUNX1 mutations had substantially different expression profiles. GSEA showed that RUNX1-mutated patients had specifically affected MYC and MAPK signaling pathways, regulation of immune response and cell death, etc. (Figure S5). The lncRNA-PCG coexpression network defined several key modules (assigned as RUNX1 modules) of similarly regulated genes that were enriched in the processes of translational regulation and RNA splicing. Based on these data, we associated Z83851.3, SNHG17, and CTD-2540L5.5 lncRNAs with RNA processing (RUNX1_1 module) and GAS5 lncRNA with protein translation (RUNX1_2 module). Interestingly, we identified an additional module (RUNX1_3 module) whose core nodes included several important genes, namely, LEF1 and RAG1 PCGs and LEF1-AS1 and TCL6 lncRNAs (Figure 3B). Enrichment analysis suggested that these genes are related to DNA repair, DNA recombination, and the p53 pathway. Table 1 summarizes enriched processes and core PCGs and lncRNAs within these modules.

### 3.5. LncRNAs Related to MDS Prognosis

One of the major goals of this work was the identification of lncRNAs linked to MDS progression potentially serving as prognostic markers of patient outcomes. By differential expression analyses, we evaluated microarray expression profiles according to (i) the overall survival (OS) of patients and (ii) their prognosis based on the IPSS-R system.

To identify genes associated with OS, we established an arbitrary cut-off to 18 months and categorized MDS/AML patients as those with short survival (deceased within 18 months, *N* = 31) or long survival (surviving more than 18 months, *N* = 25). Only 8 lncRNAs (5 upregulated/3 downregulated) and 29 PCGs (5 upregulated/24 downregulated) were significantly changed in the patients with short survival (FDR < 0.05, |logFC| > 1; Table S10). Importantly, two well-known tumorigenic lncRNAs, H19 and WT1-AS, were significantly upregulated in patients with adverse outcomes.

Then, we analyzed differential expression in MDS patients stratified according to the IPSS-R system. For the analysis, the patients were grouped into lower-risk (very low and low IPSS-R scores) and a higher-risk (high and very high IPSS-R scores) categories, while MDS with intermediate risk and AML-MRC patients were excluded from this analysis. We identified 16 lncRNAs (2 upregulated/14 downregulated) and 82 PCGs (15 upregulated/67 downregulated) with significantly changed expression in the higher-risk patients (|logFC| > 1, FDR < 0.05; Table S11). Among the lncRNAs, TCL6 and LEF1-AS were downregulated.

To explore the associations of genes related to disease progression with cellular processes, we performed GSEA on the differentially expressed genes between the lower-risk and higher-risk IPSS-R patient categories. The results showed that the most affected mechanisms include gene expression silencing through chromatin modifications, immune response, cell differentiation and proliferation, adhesion, motility, and angiogenesis (Figure S6).

The coexpression network based on these data identified modules (assigned as IPSS-R modules) that contained similarly regulated lncRNAs and PCGs. Pathway analysis further showed that the majority of these modules were related to processes involved in the immune system. However, more specific enrichment was found for IPSSR_1 module, which was associated with GO terms related to cell differentiation, growth, adhesion and migration, i.e., with the processes that may be linked with specific features of HSCs present in the BM niche. The core lncRNAs found in this module were RP11-474N8.5, RP11-879F14.2, and RP11-401P9.5. Interestingly, the expression of all the genes within the IPSSR_1 module was downregulated, suggesting that the processes of the maintenance and/or differentiation of HSCs in the BM niche may be substantially impaired in higher-risk MDS patients.

Additional interesting network modules of the IPSS-R coexpression network were IPSSR_2 and IPSSR_3 modules, both related mainly to the epigenetic modification (methylation and acetylation) and chromatin structure of DNA. Remarkably, a pair of PCG-lncRNA counterparts, LEF1 and LEF1-AS1, was found in the core of IPSSR_2 module, and TCL6 lncRNA was one of the core nodes in IPSSR_3 module, suggesting their involvement in chromatin structure in MDS. Other lncRNAs found in these modules were RP11-897M7.1 (IPSSR_2 module), ODC1-DT, LINC00963, and AC012181 (IPSSR_3 module). Table 1 summarizes enriched processes and core PCGs and lncRNAs within all these modules.

### 3.6. Individual lncRNAs as Potential Prognostic Markers of MDS

**Figure 4:**
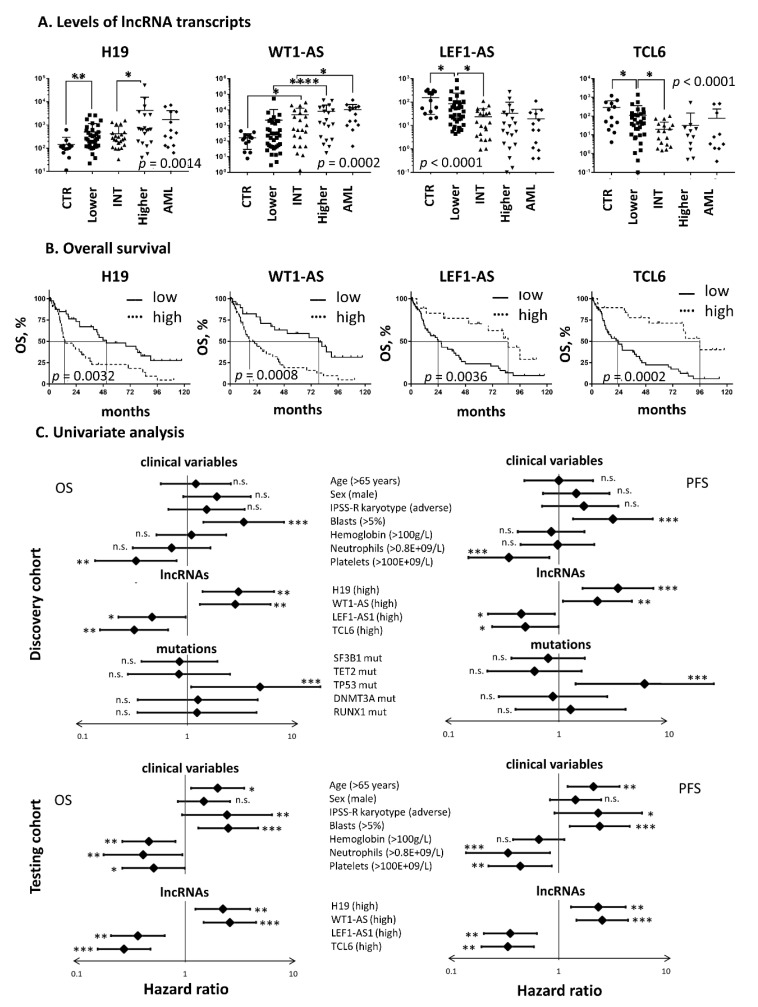
(**A**) Expression of H19, WT1-AS, TCL6, and LEF1-AS lncRNAs in CD34+ BM cells measured by RT-qPCR. The data were logarithmically scaled. The MDS patients (testing cohort) were grouped according to IPSS-R. (**B**) Kaplan-Meier curves for overall survival of MDS patients stratified based on lncRNA levels. (**C**) Forest plots of univariate analysis performed for overall survival (OS) and progression-free survival (PFS) by the log-rank test in both cohorts of MDS patients. Hazard ratios including 95% confidence intervals are plotted and the significance of the results is included (* *p* < 0.05, ** *p* < 0.01, *** *p* < 0.001, **** *p* < 0.0001, n.s.—nonsignificant). The data from mutational screening were available only for the discovery cohort.

Based on the microarray results, we chose four candidate lncRNAs applicable as prognostic biomarkers and performed a series of subsequent analyses on two independent cohorts of patients (i.e., discovery and testing cohorts; Figure S1). This set included H19, WT1-AS, TCL6, and LEF1-AS1. Initially, we reanalyzed their expression by RT-qPCR in the testing cohort and proved that the levels of all these transcripts gradually changed from healthy controls to higher-risk patients (Figure 4A), showing a strong concordance with the microarray data.

To address the question of whether the levels of these four lncRNAs are really associated with patient survival, we performed numerous log-rank tests in both cohorts of patients. Kaplan-Meier curves (Figure 4B) demonstrated a clear difference in survival between MDS patients with low vs. high levels of these lncRNAs. In both cohorts, an adverse outcome was significantly associated with high levels of H19 and WT1-AS and low levels of LEF1-AS and TCL6 (Figure 4C). Further univariate analyses examined the impact of other clinicopathological characteristics on patient survival and revealed that OS and PFS were most significantly associated with blast count, number of platelets, presence of TP53 mutation, and levels of the four lncRNAs (Figure 4C). Other variables, such as age, hemoglobin level, neutrophil count, and karyotype, also showed significant results in at least one of the tested cohorts.

To test the possible dependency between clinical factors and lncRNA alterations, we performed a multitude of Spearman correlation tests between each pair of variables (lncRNAs: H19, WT1-AS, LEF1-AS1, and TCL6; TP53 mutation; clinicopathological features: age, blast count, hemoglobin level, and numbers of neutrophils and platelets) in both cohorts of samples (Table S12). Interestingly, the percentage of marrow blasts significantly correlated with the levels of WT1-AS, TCL6, and LEF1-AS (*p* < 0.01) but was independent of the level of H19. Further, we found a strong positive correlation between the levels of LEF1-AS1 and TCL6 (*p* < 0.001), suggesting their coregulation. The presence of somatic mutations in the TP53 gene was associated with hemoglobin level (*r* = −0.316, *p* < 0.05) and WT1-AS expression (*r* = 0.445, *p* < 0.01). Interestingly, the only almost independent molecular variable was the level of the H19 transcript (with one exception of a slight negative correlation with platelet count specifically in the testing cohort; *r* = −0.254, *p* < 0.05).

To finally determine whether any of the selected lncRNAs might serve as prognostic markers for MDS outcome, we performed Cox multivariate analysis and applied the backward variable selection method to retain only the independent variables significantly contributing to the predictive power of the resulting model. Although the results from both cohorts slightly varied, the analysis revealed that predominant variables predictive of OS and PFS in MDS patients are high blast counts, high levels of the H19 transcript, and presence of somatic mutations in the TP53 gene. To a lesser extent, additional variables such as platelet count, age and TCL6 and LEF1-AS1 levels might add some prognostic value to these three major predictors (Table 2).

To stratify MDS patients based on their prognosis, the IPSS-R system is used in routine clinical practice. However, a proportion of patients scored as having lower to intermediate risk still suffer from an early progression of the disease. Therefore, we tested whether some of the selected lncRNAs can be predictive of adverse outcomes in these patients. In both cohorts, we specifically selected the patients with IPSS-R < 4.5 (i.e., lower-intermediate risk MDS patients) and reanalyzed the data using Cox multivariate regression. Neither blast count nor TP53 mutation remained informative for these patients; the only highly significant variable associated with OS and PFS was H19 expression. Additionally, some other variables such as age, platelet count, and LEF1-AS1 and TCL6 levels, were found to be predictive of patient outcome, but with less significance (Table 2).

### 3.7. Cis and Trans Correlations of Prognosis-Related lncRNAs

Perturbations of antisense RNAs can regulate the expression of their sense gene counterparts (i.e., PCGs) and vice versa via *cis*-acting regulatory elements. Therefore, we analyzed the expression of the PCG/antisense RNA pairs WT1/WT1-AS and LEF1/LEF1-AS1 by RT-qPCR in the testing cohort of samples. We found that the levels of both pairs of transcripts were highly correlated (WT1: *r* = 0.865, *p* < 0.0001; LEF1: *r* = 0.924, *p* < 0.0001) across all samples (both controls and MDS/AML-MRC patients), suggesting that their direct interactions could be necessary for WT1 and LEF1 functions (Figure S7).

Although TCL6 and H19 lncRNA do not have direct PCG counterparts, they are located in close proximity to some PCGs. TCL6 colocalizes with the TCL1A and TCL1B genes. We found only a very low level of expression for both PCGs; however, if detectable, their expression correlated with the expression of TCL6 (TCL1A: *r* = 0.645, *p* < 0.0001; TCL1B: *r* = 0.738, *p* < 0.0001; Figure S7).

**Figure 5:**
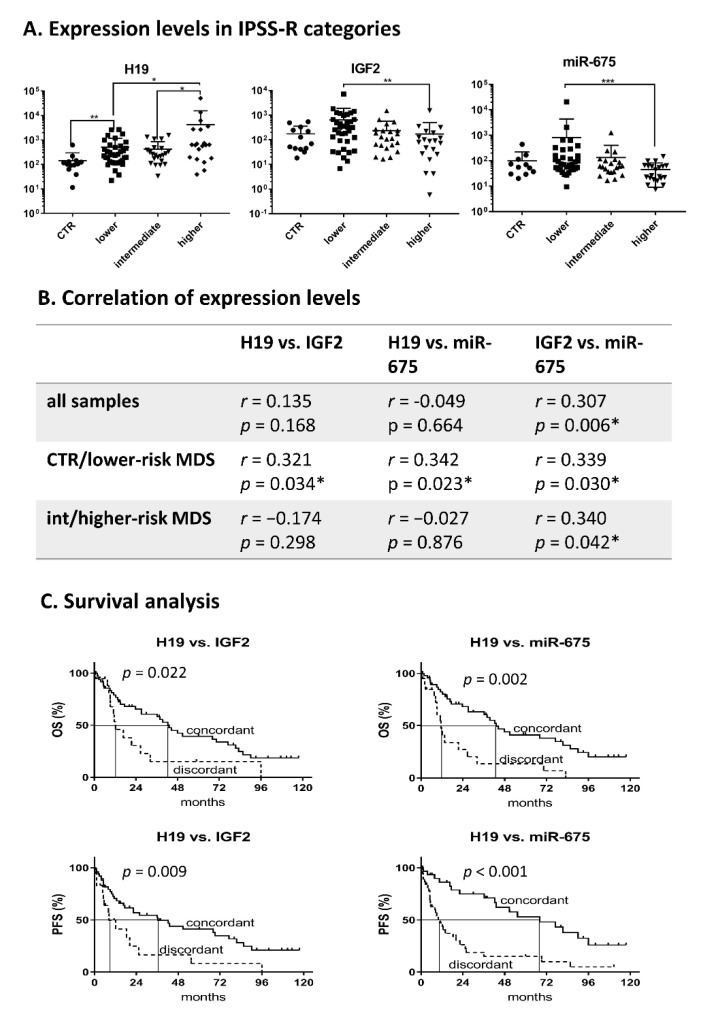
Expression of H19, IGF2, and miR-675. (**A**) Relative expression levels in MDS patients (testing cohort) grouped according to IPSS-R. (**B**) Correlation of expression levels (Spearman test) in different sample groups. (**C**) Kaplan-Meier curves for overall survival (OS) and progression-free survival (PFS) in MDS patients with concordant vs. discordant expression of the H19/IGF2 and H19/miR-675 pairs. * *p* < 0.05, ** *p* < 0.01, *** *p* < 0.001.

H19 is located adjacent to IGF2. Moreover, H19 functions as a primary template for miR-675. Therefore, we compared the expression levels of H19 to those of IGF2 and miR-675. Although we detected an upregulation of H19 and downregulation of IGF2 and miR-675 in higher-risk MDS compared to lower-risk disease (Figure 5A), the levels of H19 did not directly correlate with either IGF2 or miR-675 when analyzed in the whole cohort. However, an analysis performed separately on the samples from healthy controls and lower-risk IPSS-R patients identified a moderate correlation of H19 expression with both IGF2 (*r* = 0.321, *p* = 0.034) and miR-675 (*r* = 0.342, *p* = 0.023), whereas this concordance was disturbed in intermediate/higher-risk MDS (Figure 5B). Interestingly, the correlation between IGF2 and miR-675 remained unchanged in all samples (*r* = 0.307, *p* = 0.006; Figure 5B). Therefore, we compared the survival of patients with concordant regulation in the H19/IGF2 region with those who did not present this concordance (discordant expression was defined as a > 10-fold change in the ratio between the expression of the tested transcripts). The patients with discordant levels of H19/IGF2 or H19/miR-675 had inferior OS and PFS compared to those with concordant levels of these transcripts (univariate analyses: *p* < 0.05; Figure 5C).

**Figure 6:**
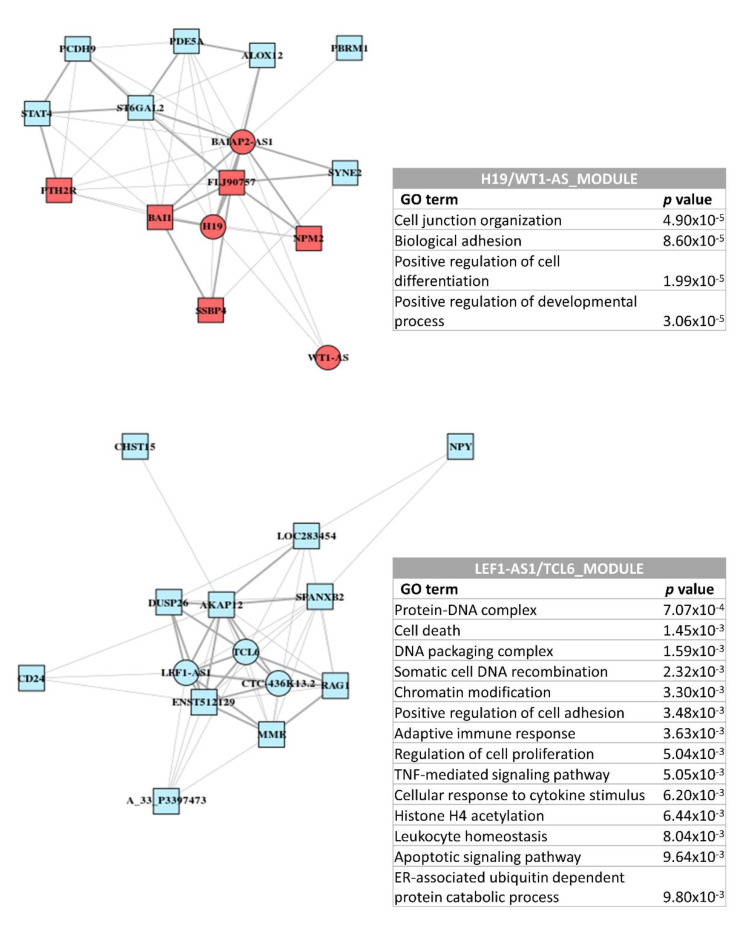
Coexpression network formed around H19, WT1-AS, LEF1-AS1, and TCL6 lncRNAs. The computational process generated two modules for the H19/WT1-AS and LEF1-AS1/TCL6 lncRNA pairs.

To address the *trans* regulatory mechanisms of the four lncRNAs related to MDS prognosis (H19, WT1-AS, LEF1-AS1, and TCL6), we constructed coexpression networks in which these lncRNAs formed the central nodes. Interestingly, the network-computing strategy generated only two modules for H19/WT1-AS and LEF1-AS1/TCL6 lncRNA pairs (assigned as the H19/WT1-AS_module and LEF1-AS1/TCL6_module, respectively), suggesting that these two pairs of genes might be functionally related. Enrichment analysis suggested that the H19/WT1-AS pair was predominantly associated with cell adhesion and differentiation processes whereas the LEF1-AS1/TCL6 pair might function in diverse mechanisms, such as chromatin modification, cytokine response, or cell proliferation and death (Figure 6).

## 4. Discussion

MDS patients display heterogeneous clinicopathological features accompanied by distinct genetic characteristics. Until recently, proteins acting through complex signaling pathways were considered exclusive vehicles linking these genomic abnormalities to clinical phenotypes. However, aside from the proteins, various classes of noncoding RN As have been shown to contribute to the variability of the disease [4,5,13,14]. In this work, we screened the lncRNA background in MDS with relation to different characteristics of the disease (i.e., diagnosis, disease subtypes, cytogenetic/mutational aberrations, and risk of progression). In addition to the description of lncRNA profiles specific for this disease, we aimed to identify lncRNAs with potential prognostic capability.

Using a genome-wide approach, we showed that samples from patients with early MDS had substantially different expression profiles than those with advanced disease. We detected the significant induction of multiple genes in early MDS, whereas there was an apparent trend of reduced gene expression along with disease progression. The major difference in the gene expression profile was surprisingly found between the MDS-EB1 and MDS-EB2 subtypes, whereas the MDS-EB2 and AML-MRC profiles were similar. Analogous observations have already been described at the miRNA level [13]. These findings suggest that leukemic transformation could be predicted by monitoring gene expression changes in the period preceding the blast count increase. This points to the issue of the accuracy of blast count cut-offs used within the diagnostic criteria. Moreover, the morphological examination of bone marrow is, to some extent, subjective and does not always reflect the true molecular background of the disease. In this context, Padron et al. [15] found that a 7.5% bone marrow blast cut-off point may discriminate the prognosis of patients with chronic myelomonocytic leukemia (CMML) with a higher resolution than with the existing 10%. Thus, clinicopathological classification criteria alone, without knowledge of molecular background, should be taken with some caution, mainly for the purpose of assessing patient prognosis and choosing appropriate treatment strategies.

Expression profiling of lncRNAs together with PCGs is a feasible approach to compare the levels of these two types of transcripts and to identify specific lncRNAs linked with pathways deregulated in a disease. Based on the combination of lncRNA and PCG data, we computationally constructed coexpression networks and identified a number of lncRNAs that might function in various cellular processes altered in MDS. Although we studied lncRNA expression in MDS patients stratified according to different aspects, we found some common features and several key lncRNAs that seem to be crucial for disease pathogenesis. The processes associated with the deregulation of gene expression primarily included immune regulation, development of blood cells, metabolism of heme, epigenetic mechanisms, RNA processing and translation. All of them have repeatedly been associated with MDS; here, we provide new data on the association of these processes with lncRNA molecules. The list of MDS-relevant lncRNAs contains H19, WT1-AS, TCL6, LEF1-AS1, EPB41L4A-AS1, PVT1, GAS5, and ZFAS1.

Although some functions of these lncRNAs have already been described in different contexts, their link to MDS pathogenesis has not yet been shown. For example, EPB41L4A-AS1 is an antisense RNA to EPB41L4A (erythrocyte membrane protein band 4.1 like 4A). This erythrocyte membrane protein is involved, via the beta-catenin pathway, in the determination of cell polarity or proliferation [16]. Here, we associated the downregulation of EPB41L4A-AS1 with ribosome formation and translational regulation in MDS. This deregulation was specifically found in MDS patients with del(5q) and can be attributed to the location of this gene in 5q22.1, near the common deleted region. Further, we detected a significant increase in PVT1 lncRNA, particularly in MDS with del(5q). PVT1 usually confers oncogenic properties on different types of cancer, including AML, and functions as a mediator of the tumor-suppressive functions of p53 [17].

Currently, the molecular testing of somatic mutations is increasingly being applied in routine practice in MDS diagnostics. The impact of somatic mutations on clinical variables and patient outcomes naturally depends on their manifestation through gene expression. To provide an understanding of how genomic variations interfere with the noncoding transcriptome, we combined data from the expression profiling with information on the mutational status of MDS patients. However, we identified only a few deregulated transcripts in the patients with vs. without particular mutations, and their numbers substantially varied among the mutations tested. SF3B1 is an RNA splicing factor, TP53 functions as a tumor suppressor inducing apoptosis, and TET2 and DNMT3A are involved in epigenetic modifications of the genome [12]; therefore, it is surprising that we detected remarkably small numbers of affected transcripts instead of a pervasive effect on the whole transcriptome. To define the reasons for this lack of expressional difference, we have to consider several aspects. A single nucleotide change in one gene might not be sufficient to induce such strong expressional change, at least when compared with the effects of del(5q) causing the haploinsufficiency of tens of genes. Moreover, diverse variants found in one gene or the cooccurrence of several mutations or cytogenetic aberrations in one patient can have variable effects on gene transcription. Therefore, larger cohorts of patients with isolated mutations are necessary to better define the transcriptional effects of somatic mutations.

RUNX1 was the gene whose mutations had the strongest transcriptional impact in our dataset, even comparable to the effects of del(5q). RUNX1 is a hematopoietic transcription factor whose somatic mutations are considered one of the most prognostically unfavorable mutational events, even in lower-risk MDS [18]. Given the adverse outcomes of RUNX1-mutated patients it is not surprising that these patients had substantially distinct expression profiles. In the patients with RUNX1 mutations, we identified the deregulation of RAG1 PCG, a core node in the DNA repair and recombination module. RAG1 (recombination activating gene 1) is an RUNX1-associated recombinase involved in antibody and T-cell receptor recombination [19]. Network modeling linked the RUNX1-RAG1 axis with LEF1/LEF1-AS and TCL6, the same transcripts whose deregulations were significantly associated with poor prognosis.

A vast body of literature exists on the deregulated expression of various PCGs and their potential applicability as prognostic markers in MDS (e.g., [20,21,22]). For example, Pellagatti et al. identified several PCGs (e.g., LEF1, CDH1, WT1, and MN1), the expression of which was significantly associated with the survival of MDS patients [20]. Transcripts of PCGs, however, are not the final effectors in the cells, unlike proteins and noncoding RNAs. Therefore, it can be assumed that lncRNA expression should be a more reliable prognostic marker than the expression of PCGs. In our study, four important lncRNAs (H19, WT1-AS, TCL6, and LEF1-AS1) were significantly associated with the outcome of MDS patients. A series of statistical tests proved that monitoring lncRNA transcription may have a highly significant potential for the prediction of outcomes in MDS patients, and the only other molecular method able to compete with them is TP53 mutational screening.

Of the four abovementioned lncRNAs, H19 (H19 imprinted maternally expressed transcript) was the most promising MDS marker in our dataset. Its increased level was associated with the rapid progression of the disease and short patient survival. Further, we associated the upregulation of H19 in higher-risk MDS with altered cell adhesion and differentiation processes in CD34+ BM cells. It has already been shown that H19 overexpression promotes leukemogenesis and predicts unfavorable prognosis in AML through its proliferative and antiapoptotic effects. Moreover, H19 overexpression correlated with a lower complete remission rate of induction therapy in AML [23]. Importantly, we showed that the level of H19 is independent of the majority of clinical and molecular variables and that its increase has strong predictive value comparable to increased blast count and the presence of TP53 mutation. Moreover, increased H19 level remained informative even in lower/intermediate-risk patients unlike blast count and TP53 mutation.

Although the H19 gene does not have a direct protein-coding counterpart, it is located in close proximity to the IGF2 gene in a region denoted as the H19/IGF2 locus. H19 and IGF2 are mutually imprinted genes, sharing one imprinting control region. In most tissues, H19 is expressed from the maternal allele, whereas IGF2 is expressed from the paternal allele [24]. H19 also functions as a primary template for miR-675, which plays an important role in tumorigenesis and the development of various cancers [25,26]. Our data suggest that the transcriptional coregulation of H19/IGF2/miR-675 seen in healthy donors and low-risk MDS becomes disrupted along with disease progression. Moreover, the discordant expression of these genes is associated with worse outcomes in MDS patients. Given to imprinting described in H19/IGF2 locus, we hypothesize that the disruption of transcriptional coregulation of H19/IGF2/miR-675 may be linked to abnormal methylation in the imprinting control region. However, this hypothesis has to be verified in an ongoing study.

Other lncRNAs whose transcription levels were strongly related to the outcomes of MDS patients were TCL6, WT1-AS, and LEF1-AS1. TCL6 (T-cell leukemia/lymphoma 6) is a lncRNA whose specific expression was initially reported in T-cell leukemia with t (14; 14) (q11; q32.1) translocation [27]. Decreased TCL6 levels have been associated with poor prognosis in patients with clear cell renal cell carcinoma [28]. WT1-AS and LEF1-AS1 are antisense transcripts of two PCGs, WT1 (Wilms tumor 1) and LEF1 (lymphoid enhancer binding factor 1), which belong among the strongest candidate genes showing an association with the prognosis of MDS patients [20]. WT1 plays a role in cell differentiation and apoptosis, and monitoring of the WT1 transcript is useful for estimating minimal residual disease and predicting outcomes in AML and MDS [29,30,31]. On the other hand, the repression of LEF1 inhibits proliferation, induces the apoptosis of CD34^+^ progenitors, and plays a critical role in the defective maturation program of myeloid progenitors [32]. Additionally, our data suggest a novel link of these two lncRNAs to chromatin modification, cytokine response, or cell proliferation and death.

Furthermore, we found that WT1-AS and LEF1-AS1 were strongly transcriptionally coregulated with their sense PCG counterparts. WT1-AS colocalizes with WT1 RNA and forms RNA:RNA duplexes, indicating a possible RNA stabilization role for WT1-AS transcripts [33]. Congrains-Castillo et al. [34] demonstrated a correlation between LEF1 and LEF1-AS1 expression in BM cells from MDS/AML patients. Upon overexpression of LEF1-AS1, they observed an inhibition of cell proliferation. However, they did not detect any alteration in LEF1 expression, suggesting that LEF1-AS1 affects cell proliferation in a LEF1-independent manner [34].

Although significantly associated with patient prognosis, the transcription of TCL6, WT1-AS, and LEF1-AS correlated with the percentage of marrow blasts. In this context, Nagasaki et al. previously reported that elevated WT1 levels may be related to increased blast cell numbers and to the presence of preleukemic MDS clones with poor prognostic chromosomal rearrangements [29]. Our correlation analyses revealed additional associations among the WT1-AS, TCL6, and LEF1-AS levels and the presence of TP53 mutations. These relations warrant that routine measurements of gene expression may be potentially confused by various reliant factors or even be redundant, at least in specific subgroups of patients (especially those with high blast counts, unfavorable cytogenetics or TP53 mutations). Thus, measurements of WT1-AS, TCL6, and LEF1-AS levels seem to provide only limited information to the current prognostic systems.

Besides description of new prognostic markers, another important aspect of this study is identification of new options for targeted therapy of the disease. Some of the examined lncRNAs, such as H19, WT1-AS, TCL6, and LEF1-AS, might serve as new druggable targets especially in higher-risk MDS. However, careful examination of functional aspects of their deregulation is required to bring necessary information for proper design of new efficient targeted therapies.

To conclude, our findings provide novel information on particular lncRNAs contributing to MDS pathogenesis and propose cellular processes associated with these transcripts. Moreover, we found that H19, WT1-AS, TCL6, and LEF1-AS1 lncRNAs are particularly associated with the outcome of MDS patients. Based on a series of statistical tests, we demonstrated that the level of H19 transcript might serve as a robust independent prognostic marker comparable to clinical variables currently used for patient stratification. Based on our data, we encourage further, larger-scale studies that will suggest a novel prognostic scoring system combining clinical variables with several genetic markers of diverse characteristics, including somatic mutations and both PCG and lncRNA expression.

## Figures and Tables

**Table 1:** Characteristics of selected modules in lncRNA-PCG coexpression networks based on differentially expressed genes between (**A**) MDS patients and healthy controls, (**B**) patients with isolated del(5q) and patients with normal karyotype, (**C**) RUNX1-mutated and RUNX1-wild type patients, and (**D**) MDS patients with lower- and higher-risk IPSS-R. Enrichment analysis was done to associate module PCGs with GO terms (*p* < 0.01). The representative core PCGs and lncRNAs with the highest module membership are listed.

DEA	Module	Associated GO Terms	Core Nodes
A. MDS vs. CTR	MDS_1	Hemoglobin complex, oxygen transport, iron ion binding, solute sodium symporter activity	PCGs: A_33_P3261428, A_33_P3328284, AC131097, ARG2, C20orf108, GABRP, LOC100128348, MOSPD1, RUNDC3A, SERPING1, SLC6A8, TRIM10
			lncRNAs: AC131097, PRKAR2A-AS1, RP4-669L17
	MDS_2	Hemoglobin complex, oxygen transport, ganglioside biosynthetic process, sialylation	PCGs: DIAPH1, EPB42, GYPB, HBD, HBBP1, OSBP2, PQLC1, SUB1, TGM2, USE1, WDFY2
			lncRNAs: CTD-2319I12, P11-640M9.1, RP11-640M9.1, RP11-558A11
	MDS_3	Golgi apparatus, protein polyubiquitination, proteasomal protein catabolic process	PCGs: AK8, C11orf2, EBPL, FBXO6, GABPB2, GLTSCR2, LGALS8, LOC644285, MS4A6A, RAB20, RPL34, THC2496362, TMEM104
			lncRNAs: AC021188, ZFAS1
	MDS_4	Translational initiation, mRNA metabolic process, cytosolic ribosome, protein localization to endoplasmic reticulum, nuclear transcribed mRNA catabolic process nonsense mediated decay, SMAD protein signal transduction, poly-A-RNA binding, cytoplasmic translation, aminoacid activation, ribonucleoprotein complex biogenesis	PCGs: A_24_P24724, ANKRD42, C11orf2, EIF4B, GEMIN5, GLTSCR2, HARS, LARS, RBM27, RPL29, RPS14, SLC26A2
			lncRNAs: CSNK1A1P1, EPB41L4A-AS1
B. Patients with isolated del(5q) vs. patients with normal karyotype	del(5q)_1	RNA binding, translational initiation, ribonucleoprotein complex, protein localization to endoplasmatic reticulum, nuclear transcribed mRNA catabolic process nonsense mediated decay, protein targeting to membrane, ribosome, large ribosomal subunit	PCGs: A_24_P59247, A_33_P3358856, DNAJC18, HARS, HNRNPA0, HSPA9, IMPDH2, MRPL45, NOA1, RPL29P2, RPS14, TLR3, ZCCHC10
			lncRNAs: EPB41L4A-AS1
	Del(5q)_2	Intracellular receptor signaling pathway, STAT cascade, positive regulation of immune system process, regulation of VEGFR signaling pathway, tyrosine phosphorylation of STAT protein, regulation of cell activation, cytokine activity, JAK/STAT cascade involved in growth hormone signaling pathway	PCGs: A_24_P143653, BRD8, C5orf56, FBXL17, FPGT, MYADM, RBM10, RNF139, SLC23A1, SPTLC1, UQCR10, YTHDC2
			lncRNAs: NUTM2A-AS1, RP11-506M13.3, STARD4-AS1
	del(5q)_3	Phosphatase inhibitor activity, platelet morphogenesis, platelet activation, hemostasis, activation of MAPK activity	PCGs: ANK1, C18orf10, CTNNBL1, DNAJC6, EPOR, KLF1, MINPP1, NMU, PCYT1B, UROD, ZFPM1
			lncRNAs: BOLA3-AS1, ENST433198.2, MIR4435-2HG
	del(5q)_4	Erythrocyte homeostasis, homeostasis of number of cells, myeloid cell differentiation, myeloid cell homeostasis, platelet morphogenesis, platelet activation	PCGs: ADAMTS14, DKNND5A, GYPB, ITLN1, LOC100128881, MFSD2B, PGF, PPAPDC1A, SPTA1, TRIB2, UBAC1
			lncRNAs: PVT1, RP11-558A11.3, RP11-797H7.5
	del(5q)_5	Oxidative phosphorylation, cellular respiration, mitochondrial envelope, organelle inner membrane, mitochondrion, mitochondrial electron transport ubiquinol to cytochrome c	PCGs: ANAPC11, ARHGAP19, CASP3, EFTUD2, FGFR1OP, GATC, GK, METTL8, NF2, PSMA6, SAE1, SENP1
			lncRNAs: OIP5-AS1, POFUT1-006
C. RUNX1-mutated vs. RUNX1-wild type patients	RUNX1_1	RNA binding, RNA processing, RNA localization, RNA splicing, termination of RNA polymerase II transcription, RNA 3’-end processing, protein sumoylation, protein folding, spliceosomal complex, cajal body	PCGs: A_33_P3268147, C11orf31, FIP1L1, FOXN2, LOC100216545, MYL12B, NEDD8, SRSF4, STK36, TMEM199, TOMM6, TPM3
			lncRNAs: CTD-2540L5.5, SNHG17, Z83851.3
	RUNX1_2	Translational initiation, RNA binding, protein localization to endoplasmatic reticulum, ribosome biogenesis, rRNA metabolic process, posttranscriptional regulation of gene expression	PCGs: A_24_P350307, A_33_P3210702, CSNK2A2, EIF3F, GNB2L1, NOA1, PAIP2, RPL3, RPL5, RPL7, RPL21, RPL22, RPS14
			lncRNAs: GAS5
	RUNX1_3	Cell cycle, immunoglobulin complex, antimicrobial humoral response, 3’ to 5’ DNA helicase activity, beta-catenin-TCF complex formation, double-strand break repair, V(D)J recombination, p53 binding, chromatin remodeling, recombinational repair, somatic cell DNA recombination, negative regulation of signal transduction by p53 class mediator	PCGs: A_33_P3397473, AKAP12, DUSP26, EBF1, LEF1, LOC283454, NPY, RAG1, SH2D4B
			lncRNAs: LEF1-AS1, LINC01013-003, TCL6
D. MDS patients with lower- vs. higher-risk IPSS-R	IPSSR_1	Locomotion, regulation of cell adhesion, blood vessel morphogenesis, regulation of cell proliferation, regulation of cell growth, positive regulation of cell differentiation, Golgi cisterna, circulatory system development, regulation of immune system process, angiogenesis, substrate-dependent cell migration, response to growth factor	PCGs: BCAR1, BTNL9, CLDN5, CLEC4G, EFNB2, GNAZ, NR2F2, NUAK1, RBP7, STAB2, TFF3
			lncRNAs: RP11-401P9.5, RP11-474N8.5, RP11-879F14.2
	IPSSR_2	Chromatin organization, immune response, chromatin silencing, negative regulation of gene expression epigenetic, regulation of cell adhesion, histone H4 acetylation	PCGs: A_24_P401601, BAI1, DLG5, LEF1, LOC116437, LOXL4, N4BP2, NPY, PTH2R, RNF19B, STAT4
			lncRNAs: LEF1-AS1, RP11-897M7.1
	IPSSR_3	Chromatin organization, histone H3K4 trimethylation, chromatin silencing, negative regulation of gene expression epigenetic, Immune system process, leukocyte homeostasis	PCGs: A_33_P3333327, CCDC106, CLEC4C, DDX60L, F11R, FLJ36777, HIST2H2AC, IFITM3, LOC100288884, SSBP4, ZNF746
			lncRNAs: AC012181, LINC00963, ODC1-DT, TCL6

**Table 2:** Multivariate Cox-regression analysis of the overall survival and progression-free survival of MDS patients. Only the variables that remained significant after backward variable selection are listed and sorted according to their descending *p*-values.

Variable	Discovery Cohort			Testing Cohort		
	HR	95% CI	*p*	HR	95% CI	*p*
A. All MDS Patients						
Overall Survival						
Blast count	19.70	3.83–101.26	<0.001	5.65	1.844–17.30	0.002
H19 level	16.92	1.73–165.12	0.015	54.35	13.10–225.62	<0.001
TP53 mutation	4.86	1.64–14.43	0.004	n.a.		
Platelet count	n.s.			0.19	0.06–0.62	0.006
LEF1-AS1 level	n.s.			0.01	0.01–0.09	0.006
TCL6 level	n.s.			104.56	1.30–8403.98	0.038
Age	27.80	1.52–507.17	0.025	n.s.		
Progression-free survival						
Blast count	7.60	1.96–29.44	0.003	7.53	2.48–22.79	<0.001
TP53 mutation	6.99	2.75–17.75	<0.001	n.a.		
H19 level	n.s.			76.13	16.97–341.42	<0.001
LEF1-AS1 level	n.s.			0.01	0.01–0.01	0.001
Platelet count	n.s.			0.13	0.03–0.50	0.003
TCL6 level	n.s.			2169.18	6.11–77.05 × 10^4^	0.010
B. Lower/intermediate-risk MDS patients (IPSS-R < 4.5)						
Overall survival						
H19 level	n.s.			14.20	3.19–64.65	0.001
TCL6 level	n.s.			0.01	0.01–0.12	0.022
Platelet count	n.s.			0.19	0.04–1.01	0.050
Age	580.70	1.39–24.28 × 10^4^	0.039	n.s.		
TP53 mutation	16.13	0.10–260.52	0.050	n.a.		
Progression-free survival						
H19 level	175.43	4.15–74.17 × 10^4^	0.007	16.37	3.79–70.67	<0.001
Age	10114.11	14.58–70.16 × 10^5^	0.006	n.s.		
Platelet count	n.s.			0.10	0.02–0.62	0.013
LEF1-AS1 level	n.s.			0.01	0.01–0.22	0.023

HR—hazard ratio, CI—confidence interval, n.s.—nonsignificant, n.a.—not analyzed.

## Supplementary Materials

The following are available online at https://www.mdpi.com/2072-6694/12/10/2726/s1, Supplementary methods, Figure S1: Stability of the selected reference genes potentially applicable for RT-qPCR normalization, Figure S2: Expression levels of PVT1, CHRM3-AS2, and EPB41LA-AS1 lncRNAs in MDS/AML-MRC patients according to their karyotype, Figure S3: Gene set enrichment analysis (GSEA) of differentially expressed PCGs in MDS/AML-MRC patients with isolated del(5q) vs. those with a normal karyotype, Figure S4: Frequency and distribution of somatic mutations in the discovery cohort, Figure S5: Gene set enrichment analysis (GSEA) of differentially expressed PCGs in MDS/AML-MRC patients with RUNX1 mutation vs. those with RUNX1 wild type, Figure S6: Gene set enrichment analysis (GSEA) of differentially expressed PCGs in MDS patients with higher- vs. lower-risk IPSS-R, Figure S7: Correlations of the expression levels of WT1 to WT1-AS, LEF1 to LEF1-AS1, and TCL6 to TCL1A/TCL1B, Table S1: Characteristics of the cohorts. The discovery cohort was examined by microarrays, and the testing cohort was used for RT-qPCR measurements, Table S2: List of significantly deregulated transcripts in MDS patients compared to healthy controls (|logFC| > 1, FDR < 0.05). Of the 83 upregulated PCGs, only the top 30 transcripts are listed, Table S3: List of significantly deregulated transcripts in AML-MRC compared to MDS patients (|logFC| > 1, FDR < 0.05). Of the 159 downregulated PCGs, only the top 30 transcripts are listed, Table S4: List of significantly deregulated transcripts in MDS/AML-MRC patients with isolated del(5q) vs. those with a normal karyotype (|logFC| > 1, FDR < 0.05). Of the 106 upregulated and 54 downregulated PCGs, only the top 30 transcripts are listed in each category, Table S5: List of significantly deregulated transcripts in MDS patients with vs. without a SF3B1 mutation (|logFC| > 0.3, FDR < 0.05), Table S6: List of significantly deregulated transcripts in MDS patients with vs. without a TET2 mutation (|logFC| > 0.3, FDR < 0.05), Table S7: List of significantly deregulated transcripts in MDS patients with vs. without a TP53 mutation (|logFC| > 0.3, FDR < 0.05), Table S8: List of significantly deregulated transcripts in MDS patients with vs. without a DNMT3A mutation (|logFC| > 0.3, FDR < 0.05), Table S9: List of significantly deregulated transcripts in MDS patients with vs. without RUNX1 mutation (|logFC| > 0.3, FDR < 0.05), Table S10: List of significantly deregulated transcripts in patients with long vs. short survival (|logFC| > 1, FDR < 0.05), Table S11: List of significantly deregulated transcripts in MDS patients with lower- vs. higher-risk IPSS-R (|logFC| > 1, FDR < 0.05), Table S12: Correlations of lncRNA expression with clinical variables of MDS patients.
