# Supplementary material for: LncRNA Profiling Reveals That the Deregulation of H19, WT1-AS, TCL6, and LEF1-AS1 Is Associated with Higher-Risk Myelodysplastic Syndrome
Source: Cancers (Basel). 2020 Sep 23;12(10):2726. doi: 10.3390/cancers12102726 (PMC7598221; doi:10.3390/cancers12102726)
Supplement: Supplementary file 1 [file cancers-12-02726-s001.pdf]

Supplementary Material: LncRNA Profiling Reveals That the Deregulation of H19, WT1-AS, TCL6, and LEF1-AS1 Is Associated with Higher-Risk Myelodysplastic Syndrome

Katarina Szikszai, Zdenek Krejcik, Jiri Klema, Nikoleta Loudova, Andrea Hrustincova, Monika Belickova, Monika Hruba, Jitka Vesela, Viktor Stranecky, David Kundrat, Pavla Pecherkova, Jaroslav Cermak, Anna Jonasova and Michaela Dostalova Merkerova

Supplementary Methods

Patients

The study included 133 patients with various subtypes of MDS, 28 patients with AML-MRC, and 22 healthy donors. The individuals were randomly divided into a discovery cohort (54 MDS patients, 14 AML-MRC patients, and 9 healthy controls) and a testing cohort (79 MDS patients, 14 AML-MRC patients, and 13 healthy controls). Bone marrow (BM) samples were obtained from the patients during routine clinical assessment at the Institute of Hematology and Blood Transfusion and the First Department of Internal Medicine, General Faculty Hospital, Prague. MDS patient age ranged from 29 to 88 years (mean 63), and the male/female distribution was 70/63. Similarly, the age of AML-MRC patients ranged from 29 to 82 years (mean 66), and the male/female distribution was 19/9. The study included only patients with no known history of previous malignancy, chemotherapy or radiation therapy. Moreover, none of the patients had received therapy for their disease or hematopoietic stem cell transplantation (HSCT) prior to BM collection. The patient's diagnoses were assessed based on the standard WHO 2016 classification criteria [1], and all the patients were classified according to the IPSS-R categories [2] except for two patients with unavailable cytogenetics. The control groups contained hematologically healthy, age matched donors (age ranged from 28 to 70 years, average 56, and male/female distribution was 14/8). Informed consent was obtained from all patients and healthy donors for inclusion in the study. The study was approved by the Institutional Scientific Board and the Local Ethics Committee and was performed in accordance with the ethical standards of the Declaration of Helsinki and its later amendments. The detailed clinical and laboratory characteristics of both cohorts, including the classification of MDS patients into subgroups, IPSS-R categories, BM features and blood counts, are summarized in Table S1.

Cell Separation and Nucleic Acid Extraction

Mononuclear cells (MNCs) were purified from BM aspirates using Ficoll-Histopaque (GE Healthcare, Munich, Germany) density centrifugation. CD34+ cells were subsequently isolated from MNCs using magnetic cell separation according to the manufacturer's instructions (Miltenyi Biotec, Bergisch Gladbach, Germany). DNA was isolated using the MagCore Genomic DNA Whole Blood Kit. Total RNA was extracted by the acid-guanidinE–phenol-chloroform method, and the samples were incubated with DNase I to prevent genomic DNA contamination. The quantity of DNA/RNA was quantified using an Invitrogen Qubit 3 Fluorometer (Thermo Fisher Scientific, Waltham, MA, USA) and the RNA integrity was assessed using an Agilent 4200 TapeStation (Agilent Technologies, Santa Clara, CA, USA).

lncRNA Microarrays and Data Analysis

GenomE-wide lncRNA profiles were determined using an Agilent Human GENCODE Custom lncRNA Expression Microarray Design v15 developed by the Bioinformatics and Genomics Group at the Centre for Genomic Regulation in Spain [3]. The array contains probes for 22,001 lncRNA

transcripts and 17,535 PCG mRNAs. The Agilent Low Input Quick Amp Labeling Kit was used for sample preparation (RNA input was set up to 200 ng) according to the manufacturer's recommendation. The hybridized arrays were scanned using an Agilent Microarray Scanner C. Microarray probes were initially mapped to the GRCh37/hg19 genome using the NovoAlign program (Novocraft Technologies, Selangor, Malaysia) and reannotated according to the UCSC Genome Browser (http://genome.ucsc.edu). Raw data were extracted using Agilent Feature Extraction Software. Quality control, quantile normalization, and filtering were performed with the Bioconductor project in the R statistical environment using the limma package. Differentially expressed lncRNAs and PCGs were identified using the empirical Bayesian method implemented in the R limma package. Multiple testing correction was performed to compute the false discovery rate (FDR) using the Benjamini-Hochberg method. To visualize the differential expression data, expression heatmaps were designed using MeV v4.3.2 software [4] and hierarchical clustering of the data was performed using average linkage and Pearson distance. The raw and normalized data have been deposited in the National Center for Biotechnology Information (NCBI) Gene Expression Omnibus (GEO) database under accession number GSE145733.

RT-qPCR

Reverse transcription quantitative PCR (RT-qPCR) was applied to measure the transcript levels of individual genes (lncRNAs: CHRM3-AS2, EPB41L4A-AS1, H19, LEF1-AS1, PVT1, TCL6, and WT1-AS; PCGs: IGF2, LEF1, WT1, TCL1A, and TCL1B; miRNAs: miR-675 and RNU48 as a reference). SuperScript IV VILO Master Mix (Thermo Fisher Scientific, Waltham, MA, USA) was used for cDNA synthesis and TaqMan gene expression assays with TaqMan universal mastermix II with UNG (Thermo Fisher Scientific) were applied for quantitative PCR using a StepOnePlus instrument (Thermo Fisher Scientific).

For the normalization of the raw C_T data of lncRNAs and PCGs, we tested several known reference genes (B2M, GAPDH, GUSB, HPRT1, TUBB, UBC, and YWHAZ). The stability of the genes was compared using the web-based tool RefFinder that integrates four major currently available computational programs (geNorm, Normfinder, BestKeeper, and the comparative delta-Ct method) [5]. Based on the results from this optimization procedure (Figure S1), the RT-qPCR data were finally normalized to the HPRT1 reference gene and further processed by the $2^{-\Delta\Delta CT}$ method [6].

Mutational Screening and Data Analysis

The TruSight Myeloid Sequencing Panel Kit (Illumina, San Diego, CA, USA) containing 568 amplicons in 54 genes associated with myeloid malignancies was used for the mutational screening of patients from the discovery cohort. The amplicon library was constructed according to the manufacturer's recommendations. After library purification and subsequent normalization on beads, quantification was performed by the Kapa Library Quantification Kit (Kapa Biosystems, Wilmington, MA, USA). The libraries were pooled and 2x150 bp paired-end sequenced with Rapid SBS Kit V2 chemistry on an Illumina HiSeq 2500 instrument. FASTQ files were subjected to initial quality control by FastQC. Adaptor trimming was performed by Trimmomatic, and low-quality sequences were removed by Illuminaclip. The remaining reads were aligned to the human genome hg19 using BWA-MEM. Variants were detected by LoFreq v2.1.3.1. and annotated using Variant Effect Predictor (Ensembl). The clinical significance of each variant was verified in several genomic databases (UCSC, COSMIC, ExAC, and PubMed). The arbitrary cut off was set at 5% of variant allele frequency (VAF).

Statistical Analysis

Statistical analyses were performed using GraphPad Prism 7 (GraphPad Software, La Jolla, CA, USA) and SPSS software (IBM, Armonk, NY, USA). A nonparametric Mann-Whitney test was used to compare transcript levels and clinical parameters between two groups of samples. The Spearman rank test was performed to assess the correlation of continuous variables. The survival distributions for overall survival (OS) and progression-free survival (PFS) were estimated using the Kaplan-Meier

method, and the differences were compared using the log-rank test. For the determination of the optimum cut-off values of transcript levels, we computed the p-values with the log-rank test on a dense net local computation and defined the cut-off points using Gaussian mixture models where the obtained *p*-values were divided into two components. For multivariate analysis, we estimated a Cox proportional hazards regression model with the Min-Max method for normalizing the data. The backward likelihood method was applied for the reduction of variables. The differences were considered statistically significant when $p < 0.05$.

Pathway Analysis

The functional changes related to deregulations in gene expression were assessed using gene set enrichment analysis (GSEA) [7]. As a reference, c2 (c2.all.v7.0.symbols.gmt [Curated]), c5 (c5.all.v7.0.symbols.gmt [Gene Ontology, GO]), and hallmark (h.all.v7.0.symbols.gmt [Hallmarks]) gene sets from the Molecular Signatures Database were utilized. The number of permutations was set to 1,000. The enrichment results with $p < 0.05$ were further considered.

LncRNA-PCG Coexpression Networks

The network analysis directly stems from the network-based lncRNA module functional annotation method introduced by Liu et al. [8]. First, we identified differentially expressed lncRNAs and PCGs (FDR < 0.05) and constructed a correlation matrix for these transcripts. The correlation was calculated for all the lncRNA-PCG pairs, and the absolute value of the Pearson correlation coefficient represented each pair. Second, non-negative matrix factorization (NMF) was used to extract modules from the correlation matrix. In particular, standard factorization based on the Kullback-Leibler divergence was employed [9]. The factorization was run multiple times for different numbers of modules and with different random seeds to compute the initial values for the factor matrices to avoid improper local minima of the objective function. The Frobenius norm of the factorization residual matrix served as the factorization objective function. Then, each module was functionally annotated. All the PCGs were mapped to the corresponding GO terms, and the terms with at least two corresponding PCGs were kept. GO enrichment analysis served to annotate individual modules, and Fisher's exact test was used to calculate the score for the individual terms. Eventually, the representative cores of the individual modules were plotted. In each plot, 4 lncRNAs and 13 PCGs with the highest module membership visually represented the module. Edges connect those nodes whose absolute correlation exceeds the median module correlation (weak) and its third quartile (strong). The GO terms with $p < 0.01$ associated with these modules are listed in the output tables. The network analysis was carried out in the R statistical environment with the limma, NMF, GSEABase and iGraph packages.

Supplementary Figures and Tables

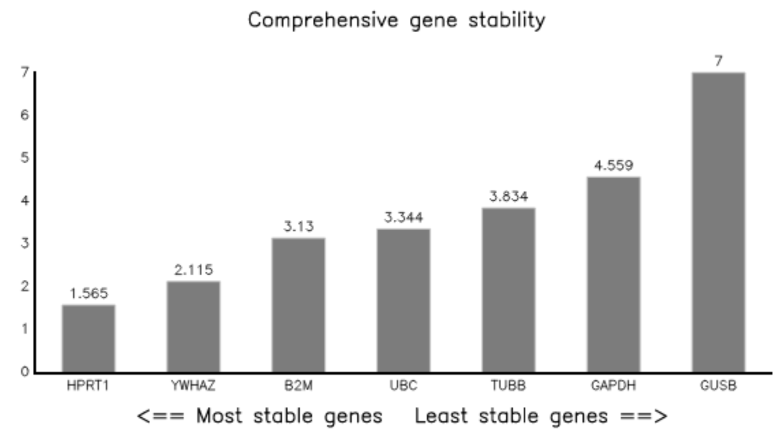

	Ranking Order						
**Method**	**1**	**2**	**3**	**4**	**5**	**6**	**7**
**Delta CT**	**HPRT1**	**YWHAZ**	**TUBB**	**B2M**	**UBC**	**GAPDH**	**GUSB**
**BestKeeper**	**UBC**	**B2M**	**GAPDH**	**TUBB**	**YWHAZ**	**HPRT1**	**GUSB**
**Normfinder**	**HPRT1**	**YWHAZ**	**TUBB**	**B2M**	**UBC**	**GAPDH**	**GUSB**
**Genorm**	**HPRT1 | YWHAZ**		**B2M**	**GAPDH**	**UBC**	**TUBB**	**GUSB**
**Recommended ranking**	**HPRT1**	**YWHAZ**	**B2M**	**UBC**	**TUBB**	**GAPDH**	**GUSB**

Figure S1. Stability of the selected reference genes potentially applicable for RT-qPCR normalization. The tested genes (B2M, GAPDH, GUSB, HPRT1, TUBB, UBC, and YWHAZ) were ranked using the web-based tool RefFinder, which integrates four major currently available computational programs (geNorm, Normfinder, BestKeeper, and the comparative delta-Ct method). Based on the rankings from each program, RefFinder assigns an appropriate weight to an individual gene and calculates the geometric mean of their weights for the overall final ranking [5].

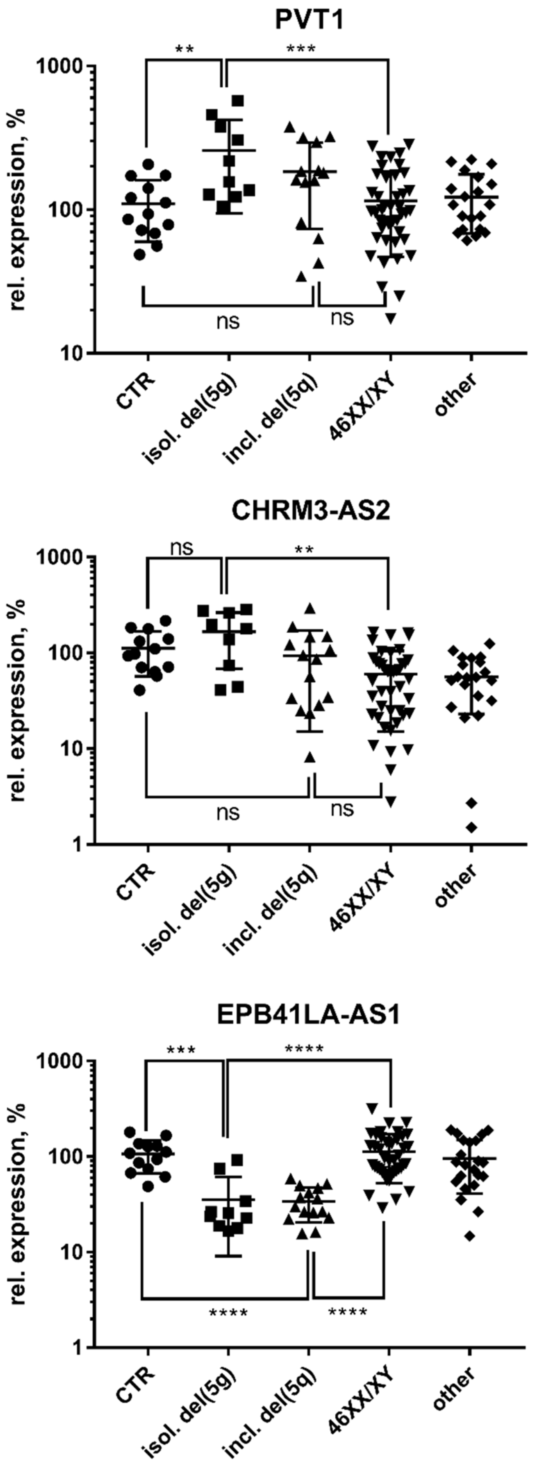

Figure S2. Expression levels of PVT1, CHRM3-AS2, and EPB41LA-AS1 lncRNAs in MDS/AML-MRC patients according to their karyotype. Relative expression was assessed by RT-qPCR. CTR-healthy controls, ** $p < 0.01$, *** $p < 0.001$, **** $p < 0.0001$, ns-nonsignificant.

GSEA: del(5q) patients *vs.* normal karyotype patients

Gene sets	NES	*P*
Enriched in del(5q) patients (the top 8 gene sets)		
STAT5 targets DN (Wierenga)	2.79	<0.001
Hematopoietic stem cell DN (Jaatinen)	2.71	<0.001
AML of FAB M7 type (Ross)	2.59	<0.001
Progenitor (Eppert)	2.54	<0.001
Platelet activation, signaling, and aggregation (Reactome)	2.52	<0.001
CML quiescent vs. normal quiescent UP (Graham)	2.46	<0.001
Mammary stem cell UP (Lim)	2.43	<0.001
Heme metabolism (Hlmark)	2.42	<0.001
Enriched in patients with normal karyotype		
Tamoxifen resistance DN (Massarweh)	−1.87	<0.001
3′-UTR mediated translational regulation (Reactome)	−2.15	0.003
Translational initiation (GO)	−1.99	0.003
Ribosomal subunit (GO)	−1.95	0.003
Translation (Reactome)	−2.03	0.008
Nuclear transcr. mRNA catabolic process nonsense mediated decay (GO)	−1.83	0.011
Hematopoietic stem cell UP (Jaatinen)	−1.82	0.011
Ribosome (GO)	−1.80	0.015

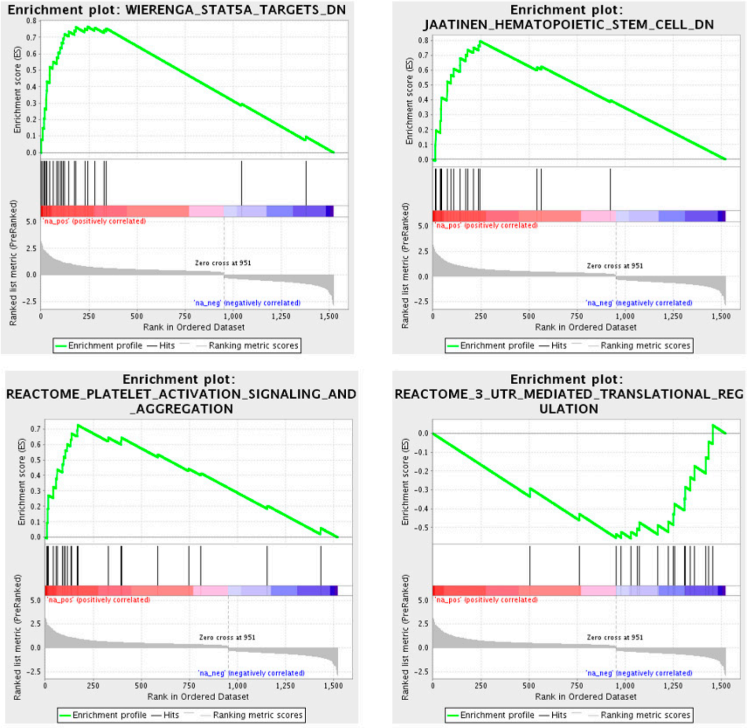

Figure S3. Gene set enrichment analysis (GSEA) of differentially expressed PCGs in MDS/AML-MRC patients with isolated del(5q) vs. those with a normal karyotype. Four selected enrichment plots are shown. NES -normalized enrichment score. References: Wienerga et al. [10], Jaatinen et al. [11], Ross et al. [12], Eppert et al. [13], Graham et al. [14], Lim et al. [15], and Massarweh et al. [16].

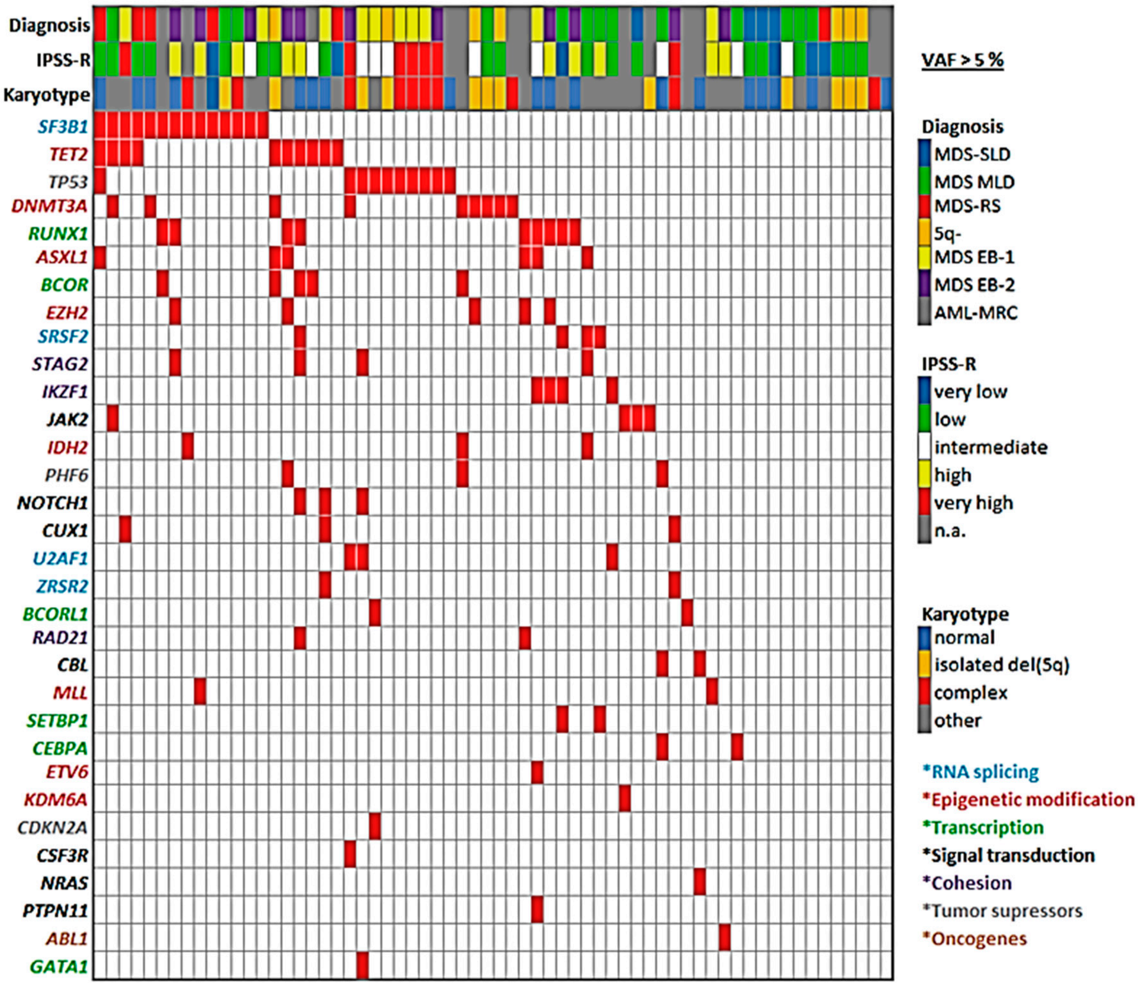

Figure S4. Frequency and distribution of somatic mutations in the discovery cohort.

GSEA: MDS patients with *vs.* without *RUNX1* mutation

Gene sets	NES	*p*
Enriched in RUNX1-mutated MDS		
Golgi apparatus part (GO)	2.23	<0.001
Vesicle membrane (GO)	1.77	0.009
MYC targets V1 (Hallmark)	1.63	0.019
Response to GSK3 inhibitor SB216763 UP (Wang)	1.71	0.025
Protein phosphorylation (GO)	1.54	0.039
mRNA metabolic process (GO)	1.48	0.044
Enriched in RUNX1-wildtype MDS		
B lymphocyte progenitor (Haddad)	−1.91	0.001
MAPK8 targets UP (Yoshimura)	−1.80	0.001
Tretinoin response UP (Martens)	−1.79	0.001
Immune response (GO)	−1.68	0.005
Sequence specific DNA binding (GO)	−1.62	0.012
Immune system development (GO)	−1.62	0.018
Positive regulation of cell death (GO)	−1.57	0.023
HDAC targets silenced by methylation UP (Heller)	−1.58	0.030

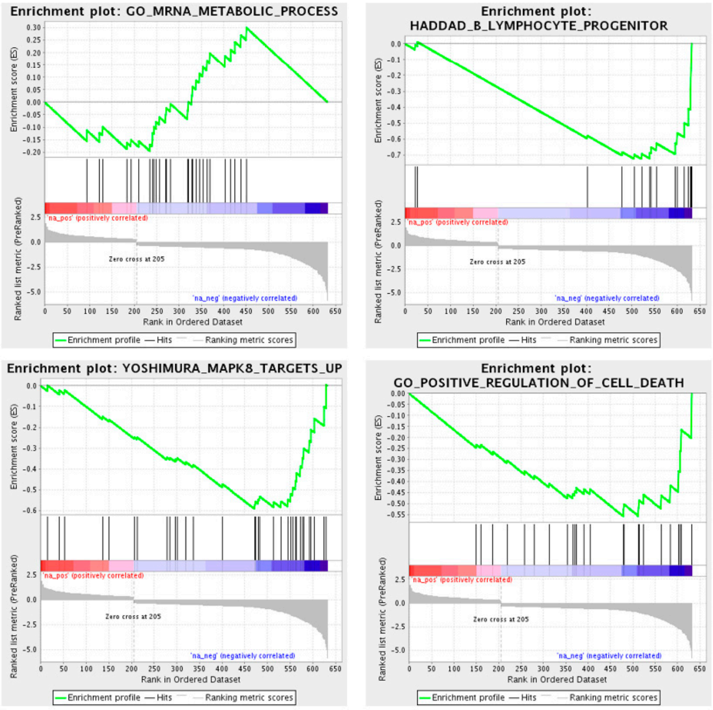

Figure S5. Gene set enrichment analysis (GSEA) of differentially expressed PCGs in MDS/AML-MRC patients with RUNX1 mutation vs. those with RUNX1 wild type. Four selected enrichment plots are shown. NES—normalized enrichment score. References: Wang et al. [17], Haddad et al. [18], Yoshimura et al. [19], Martens et al. [20], and Heller et al. [21].

GSEA: lower- *vs.* higher-risk MDS

Gene sets	NES	*p*
Enriched in higher-risk MDS		
Response to GSK3 inhibitor SB216763 UP (Wang)	1.71	0.019
MEF HCP with H3K27ME3 (Mikkelsen)	1.67	0.020
NPC HCP with H3 unmethylated (Meissner)	1.51	0.030
Chronic myelogenous leukemia UP (Diaz)	1.39	0.045
Enriched in lower-risk MDS (the top 10 gene sets)		
B-lymphocyte progenitor (Haddad)	−2.21	<0.001
Angioimmunoblastic lymphoma UP (Piccaluga)	−2.04	<0.001
Hematopoietic stem cell DOWN (Jaatinen)	−1.95	<0.001
Blood vessel morphogenesis (GO)	−1.86	<0.001
Positive regulation of cell differentiation (GO)	−1.84	<0.001
Regulation of response to external stimulus (GO)	−1.81	<0.001
Immune response (GO)	−1.80	<0.001
Targets of MLL/AF9 fusion (Kumar)	−1.84	0.001
Locomotion (GO)	−1.83	0.001
Perulation of cell adhesion (GO)	−1.76	0.001

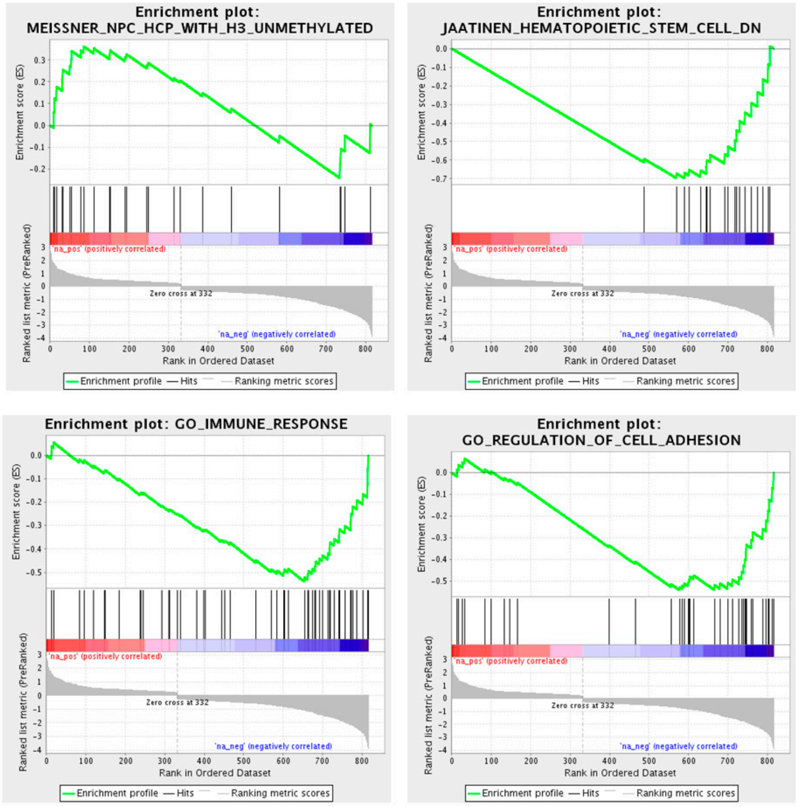

Figure S6. Gene set enrichment analysis (GSEA) of differentially expressed PCGs in MDS patients with higher- vs. lower-risk IPSS-R. Four selected enrichment plots are shown. NES-normalized enrichment score. References: Wang et al. [17], Mikkelsen et al. [22], Meissner et al. [23], Diaz et al. [24], Haddad et al. [18], Piccaluga et al. [25], Jaatinen et al. [11], and Kumar et al. [26].

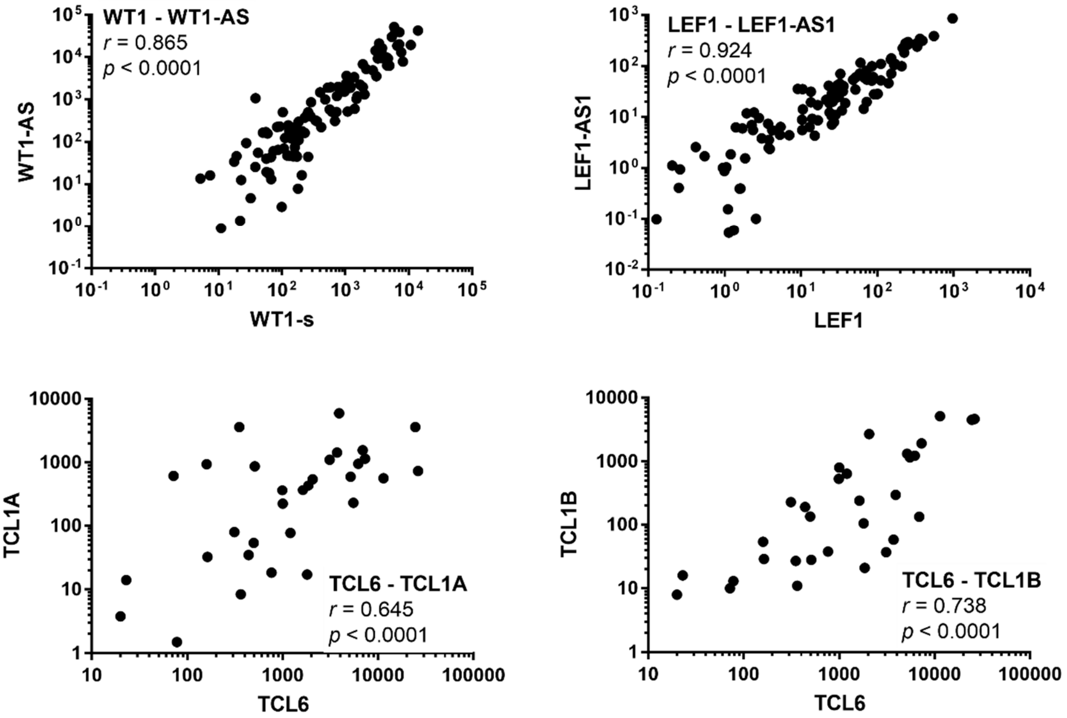

Figure S7. Correlations of the expression levels of WT1 to WT1-AS, LEF1 to LEF1-AS1, and TCL6 to TCL1A/TCL1B.

Table S1. Characteristics of the cohorts. The discovery cohort was examined by microarrays, and the testing cohort was used for RT-qPCR measurements.

Variable	Discovery Cohort	Testing Cohort
Number of samples (healthy controls/MDS/AML-MRC)	77 (9/54/14)	106 (13/79/14)
Healthy controls	9	13
Gender (male/female)	6/3	8/5
Age, mean (range)	61 (45–72)	52 (28–70)
MDS	54	79
Diagnosis (SLD/MLD/RS-SLD/RS-MLD/5q-/EB1/EB2)	5/13/4/3/7/10/12	8/12/8/8/12/10/21
Sex (male/female)	29/25	41/38
Age: mean (range)	65 (31–82)	62 (29–88)
IPSS-R category (very low/low/intermediate/high/very high/n.a.)	7/19/9/10/9/0	12/25/21/11/8/2
IPSS-R karyotype (very good/good/intermediate/poor/very poor/n.a.)	2/36/6/2/8/0	0/65/4/1/7/2
Cytogenetic features		
normal karyotype	16	38
isolated del(5q)	12	10
isolated del(20q)	4	4
isolated +8	2	4
complex	8	7
other	13	14
n.a.	0	2
Somatic mutations		
no. of patients with detected mutations (%)	39 (76%)	
no. of mutations/patient: 0/1/2/3/4/5/6/7/n.a.	12/17/7/3/8/3/0/1/3	n.a.
Marrow blasts [%]: mean (range)	5.6 (0.0–19.0)	6.1 (0.2–19.4)
Hemoglobin (g/L): mean (range)	99 (68–159)	98 (67–139)
Neutrophils ($x10^9$/L): mean (range)	2.3 (0.1–11.4)	2.4 (0.1–8.0)
Platelets ($x10^9$/L): mean (range)	184 (26–597)	205 (19–766)
Follow-up, number of patients	53	79
mean follow-up [months] (range)	26 (1–115)	34 (1–118)
i. HSCT (censored), number of patients	2	13
mean time to HSCT [months] (range)	16 (5–27)	18 (1–59)
ii. progression, number of patients	33	50
mean time to progression [months] (range)	23 (1–55)	25 (1–90)
iii deceased, number of patients	28	48
mean time to death [months] (range)	21 (1–78)	30 (1–96)
iv. alive (censored), number of patients	23	18
mean follow-up time [months] (range)	34 (1–115)	60 (1–118)
AML-MRC	14	14
Sex (male/female)	12/2	7/7
Age: mean (range)	69 (58–77)	63(29–82)
Cytogenetic features		
normal karyotype	4	6
isolated del(5q)	1	0
isolated +8	3	1
complex	3	3
other	3	5

Somatic mutations<br>no. of patients with detected mutations (%)<br>no. of mutations/patient: 0/1/2/3/4/na	 11 (85%)<br>2/4/4/1/2/1	n.a.
Marrow blasts [%]: mean (range)	26.5 (20.0–33.0)	35.0 (20.0–77.0)
Hemoglobin (g/L): mean (range)	97 (78–114)	97 (70–132)
Neutrophils (x10^9/L): mean (range)	1.7 (0.06–11.8)	2.1 (0.1–15.7)
Platelets (x10^9/L): mean (range)	93 (13–578)	59 (5–196)

n.a.—not analyzed.

Table S2. List of significantly deregulated transcripts in MDS patients compared to healthy controls (|logFC| > 1, FDR < 0.05). Of the 83 upregulated PCGs, only the top 30 transcripts are listed. logFC—binary logarithm of fold change, FDR—false discovery rate.

No.	Transcript	Chromosome	logFC	FDR
lncRNAs upregulated in MDS				
1	PRKAR2A-AS1	chr3	3.78	2.68×10^{-3}
2	RP11-408E5.5	chr13	3.22	3.29×10^{-4}
3	H19	chr11	3.18	2.08×10^{-3}
4	RP5-867C24.4	chr17	2.75	2.75×10^{-2}
5	EMCN-IT1	chr4	2.58	1.41×10^{-5}
6	RP11-558A11.3	chr16	2.30	5.01×10^{-6}
7	LINC00570	chr2	2.16	1.41×10^{-2}
8	WT1-AS	chr11	2.13	4.51×10^{-2}
9	RP11-677I18.3	chr11	1.94	3.57×10^{-4}
10	RP11-567J20.3	chr8	1.82	3.08×10^{-2}
11	RP11-753D20.1	chr14	1.77	1.15×10^{-2}
12	LINC00640	chr14	1.66	4.90×10^{-2}
13	FAM225A	chr9	1.61	1.51×10^{-2}
14	AC131097.3	chr2	1.51	1.90×10^{-2}
15	RP4-669L17.2	chr1	1.48	2.59×10^{-3}
16	MEG8	chr14	1.36	1.70×10^{-2}
17	AL132709.8	chr14	1.28	3.26×10^{-2}
18	CTD-2373N4.5	chr8	1.23	1.33×10^{-2}
19	RP11-792D21.2	chr4	1.22	3.29×10^{-4}
20	RP5-1029F21.4	chr17	1.19	3.55×10^{-2}
21	AC017076.5	chr2	1.12	3.25×10^{-2}
22	PVT1	chr8	1.12	3.67×10^{-2}
23	CTD-2319I12.2	chr17	1.11	5.66×10^{-4}
24	RP11-277L2.4	chr1	1.09	4.39×10^{-2}
25	AL132709.5	chr14	1.08	2.15×10^{-2}
26	AC020571.3	chr2	1.08	2.95×10^{-2}
27	RP1-249H1.4	chr6	1.08	2.12×10^{-2}
28	LINC00484	chr9	1.04	1.58×10^{-2}
lncRNAs downregulated in MDS				
1	U3	chr18	-1.40	4.96×10^{-4}
2	AC079779.4	chr2	-1.18	2.55×10^{-2}
3	ST6GAL2-IT1	chr2	-1.03	3.98×10^{-2}
4	RP11-13K12.1	chr17	-1.01	4.12×10^{-2}
PCGs upregulated in MDS				
1	HBG1	chr11	5.45	1.45×10^{-5}
2	HBBP1	chr11	4.00	4.70×10^{-5}
3	GYPB	chr4	3.93	4.70×10^{-5}
4	PGF	chr14	3.41	2.08×10^{-5}
5	IFI27	chr14	3.36	1.44×10^{-3}
6	OAS1	chr12	3.15	2.24×10^{-2}
7	NCAM1	chr11	3.15	9.79×10^{-3}
8	SH2D1A	chrX	2.98	8.45×10^{-3}
9	GYPB	chr4	2.93	2.95×10^{-7}
10	HBA2	chr16	2.91	2.83×10^{-2}
11	TMCC2	chr1	2.80	1.79×10^{-3}
12	TRIM10	chr6	2.78	1.51×10^{-4}
13	SPAG6	chr10	2.73	2.90×10^{-2}
14	ALDH1A3	chr15	2.64	2.24×10^{-2}
15	EPB42	chr15	2.53	4.30×10^{-2}
16	SRMS	chr20	2.43	1.33×10^{-2}
17	C20orf108	chr20	2.39	1.25×10^{-3}
18	SLC6A9	chr1	2.39	4.75×10^{-3}

19	BAI1	chr8	2.36	1.77×10^{-4}
20	PABPC4L	chr4	2.32	1.53×10^{-2}
21	LOC285758	chr6	2.28	4.70×10^{-5}
22	FHDC1	chr4	2.28	2.17×10^{-2}
23	ARG2	chr14	2.24	1.45×10^{-2}
24	SLC6A8	chr16	2.08	2.51×10^{-2}
25	OSBP2	chr22	1.89	1.89×10^{-3}
26	RFPL4A	chr19	1.88	2.32×10^{-2}
27	ABCC13	chr21	1.80	2.32×10^{-2}
28	IL2RA	chr10	1.80	2.70×10^{-3}
29	ENST00000515150	chr4	1.78	4.67×10^{-4}
30	LOC284561	chr1	1.78	3.17×10^{-2}
PCGs downregulated in MDS				
1	ECEL1P2	chr2	−1.81	1.26×10^{-3}
2	A_33_P3258324	chr19	−1.34	4.58×10^{-2}
3	AVP	chr20	−1.21	1.17×10^{-2}
4	HLF	chr17	−1.08	1.29×10^{-3}

Table S3. List of significantly deregulated transcripts in AML-MRC compared to MDS patients (|logFC| > 1, FDR < 0.05). Of the 159 downregulated PCGs, only the top 30 transcripts are listed. logFC—binary logarithm of fold change, FDR—false discovery rate, ns—nonsignificant transcript.

No.	Transcript	Chromosome	logFC	FDR
lncRNAs upregulated in AML-MRC				
ns	ns	ns	ns	ns
lncRNAs Downregulated in AML-MRC				
1	AC004510.3	chr19	−3.28	1.65×10^{-5}
2	RP11-489D6.2	chr15	−2.45	4.60×10^{-3}
3	VPS9D1-AS1	chr16	−2.15	8.63×10^{-4}
4	RP11-96B2.1	chr8	−2.01	8.63×10^{-4}
5	RP11-327I22.8	chr9	−1.98	3.80×10^{-2}
6	PVT1	chr8	−1.86	1.78×10^{-2}
7	RP11-48O20.4	chr1	−1.52	2.74×10^{-2}
8	RP11-315A17.1	chr4	−1.39	1.22×10^{-2}
9	CTD-2319I12.2	chr17	−1.34	1.22×10^{-2}
10	CXADRP3	chr18	−1.25	7.21×10^{-5}
11	C1QTNF9B-AS1	chr13	−1.07	2.36×10^{-2}
PCGs upregulated in AML-MRC				
1	LATS2	chr13	1.07	1.57×10^{-2}
2	GUCY1A3	chr4	1.04	3.41×10^{-2}
PCGs downregulated in AML-MRC				
1	DEFA3	chr8	−3.72	1.34×10^{-2}
2	PRG3	chr11	−3.64	7.85×10^{-3}
3	C21orf67	chr21	−3.44	3.15×10^{-2}
4	RBP7	chr1	−3.19	1.12×10^{-3}
5	IGHV1-18	chr14	−3.15	1.88×10^{-2}
6	IGKV4-1	chr2	−3.15	3.90×10^{-2}
7	IGLV3-10	chr22	−3.06	3.58×10^{-2}
8	SLC10A4	chr4	−2.87	1.66×10^{-3}
9	STAB2	chr12	−2.85	7.07×10^{-3}
10	ST6GALNAC1	chr17	−2.83	1.92×10^{-3}
11	DUSP26	chr8	−2.72	3.49×10^{-2}
12	APOC1	chr19	−2.67	1.00×10^{-2}
13	NMU	chr4	−2.62	1.57×10^{-2}
14	SELENBP1	chr1	−2.62	2.49×10^{-2}
15	EPB42	chr15	−2.55	5.00×10^{-2}
16	IGHV1-2	chr14	−2.53	2.75×10^{-2}
17	CLEC4G	chr19	−2.51	9.99×10^{-4}
18	SEC14L4	chr22	−2.50	7.85×10^{-3}
19	C10orf116	chr10	−2.49	6.34×10^{-3}
20	EPX	chr17	−2.47	1.28×10^{-2}
21	SPAG6	chr10	−2.45	3.87×10^{-2}
22	GYPB	chr4	−2.42	2.75×10^{-2}
23	ANK1	chr8	−2.40	1.34×10^{-2}
24	COL6A5	chr3	−2.38	7.07×10^{-3}
25	CYP1B1	chr2	−2.37	1.57×10^{-2}
26	GTSF1	chr12	−2.37	2.96×10^{-2}
27	AKR1C1	chr10	−2.37	8.07×10^{-3}
28	SNX22	chr15	−2.37	5.26×10^{-4}
29	KLF1	chr19	−2.35	7.28×10^{-3}
30	CHI3L1	chr1	−2.33	3.55×10^{-2}

Table S4. List of significantly deregulated transcripts in MDS/AML-MRC patients with isolated del(5q) vs. those with a normal karyotype (|logFC| > 1, FDR < 0.05). Of the 106 upregulated and 54 downregulated PCGs, only the top 30 transcripts are listed in each category. logFC—binary logarithm of fold change, FDR—false discovery rate.

No.	Transcript	Chromosome	logFC	FDR
lncRNAs upregulated in patients with isolated del(5q)				
1	EMCN-IT1	chr4	3.01	4.60×10^{-3}
2	RP11-56F10.3	chr9	2.27	3.11×10^{-3}
3	AC026806.2	chr19	2.26	2.91×10^{-2}
4	RP11-185E8.1	chr3	2.07	3.58×10^{-2}
5	RP11-264B17.5	chr16	2.06	3.23×10^{-2}
6	CTB-114C7.3	chr5	1.93	1.49×10^{-2}
7	CHRM3-AS2	chr1	1.88	6.58×10^{-4}
8	MAST4-IT1	chr5	1.78	2.66×10^{-2}
9	RP4-773A18.4	chr1	1.70	2.49×10^{-2}
10	RP3-510D11.2	chr1	1.59	1.94×10^{-2}
11	RP11-496N12.6	chr1	1.56	3.16×10^{-2}
12	RP11-797H7.5	chr7	1.38	4.88×10^{-2}
13	RP11-83N9.5	chr9	1.31	6.56×10^{-3}
14	PVT1	chr8	1.22	3.52×10^{-2}
15	CCDC26	chr8	1.10	4.31×10^{-2}
16	LINC00534	chr8	1.04	3.80×10^{-2}
lncRNAs downregulated in patients with isolated del(5q)				
1	TTN-AS1	chr2	−3.11	5.06×10^{-3}
2	RP11-171I2.4	chr2	−1.42	9.60×10^{-3}
3	RP11-861E21.1	chr18	−1.30	3.47×10^{-2}
4	RP11-434C1.1	chr12	−1.22	3.78×10^{-2}
5	ZFAS1	chr20	−1.18	9.61×10^{-3}
6	STARD4-AS1	chr5	−1.15	2.81×10^{-2}
7	RP1-69M21.2	chr1	−1.14	5.06×10^{-3}
8	EPB41L4A-AS1	chr5	−1.10	8.31×10^{-6}
9	CTC-345K18.2	chr5	−1.09	6.58×10^{-4}
10	AC116366.5	chr5	−1.05	9.61×10^{-3}
11	GAS5	chr1	−1.04	3.10×10^{-2}
12	CTC-304I17.3	chr17	−1.03	2.71×10^{-2}
13	PCBP1-AS1	chr2	−1.02	3.16×10^{-2}
14	RP11-493K19.3	chr3	−1.02	4.02×10^{-2}
15	RP11-169D4.2	chr11	−1.02	2.85×10^{-2}
PCGs upregulated in patients with isolated del(5q)				
1	HBBP1	chr11	5.07	1.59×10^{-2}
2	CNN1	chr19	3.09	1.81×10^{-2}
3	SLC35D3	chr6	2.95	1.67×10^{-2}
4	SPOCD1	chr1	2.79	1.27×10^{-2}
5	TMEM158	chr3	2.79	2.49×10^{-2}
6	ENST00000515150	chr4	2.70	3.57×10^{-3}
7	LAT	chr16	2.57	1.05×10^{-2}
8	PLIN2	chr9	2.48	5.53×10^{-3}
9	CLEC1B	chr12	2.40	4.16×10^{-2}
10	CD40LG	chrX	2.39	3.40×10^{-3}
11	SPAG6	chr10	2.39	1.78×10^{-2}
12	GNAZ	chr22	2.37	1.24×10^{-2}
13	THBS1	chr15	2.36	4.41×10^{-2}

14	ST6GALNAC1	chr17	2.26	2.22×10^{-3}
15	LGALS12	chr11	2.25	3.81×10^{-3}
16	LY6G6F	chr6	2.23	2.41×10^{-2}
17	CTTN	chr11	2.22	2.91×10^{-2}
18	LRRC32	chr11	2.22	4.15×10^{-2}
19	NRGN	chr11	2.14	4.72×10^{-2}
20	TUBAL3	chr10	2.13	6.38×10^{-3}
21	VSTM1	chr19	2.11	3.57×10^{-3}
22	CATSPER1	chr11	2.09	4.71×10^{-2}
23	DENND2C	chr1	2.08	3.42×10^{-2}
24	COL6A5	chr3	2.04	1.12×10^{-2}
25	TMEM40	chr3	2.00	3.68×10^{-2}
26	ACE2	chrX	2.00	2.91×10^{-2}
27	SLC2A14	chr12	1.99	1.15×10^{-2}
28	TUBA4A	chr2	1.95	2.83×10^{-2}
29	RAB6B	chr3	1.91	4.22×10^{-3}
30	PPAPDC1A	chr10	1.89	1.20×10^{-2}
PCGs downregulated in the patients with isolated del(5q)				
1	ANK3	chr10	−2.78	3.69×10^{-2}
2	APBB2	chr4	−2.16	3.83×10^{-2}
3	THC2753069	chr17	−2.08	4.54×10^{-2}
4	EGR1	chr5	−1.90	5.16×10^{-3}
5	USP9Y	chrY	−1.85	4.89×10^{-2}
6	ENST00000507296	chr8	−1.79	2.29×10^{-2}
7	NCRNA00185	chrY	−1.72	2.56×10^{-2}
8	ELFN1	chr7	−1.71	4.46×10^{-2}
9	ETV7	chr6	−1.68	1.33×10^{-2}
10	C17orf51	chr17	−1.62	3.92×10^{-2}
11	PLEKHG5	chr1	−1.61	2.40×10^{-2}
12	SLC23A1	chr5	−1.42	4.16×10^{-2}
13	C5orf56	chr5	−1.41	1.51×10^{-3}
14	EN2	chr7	−1.41	4.12×10^{-2}
15	AK125205	chr2	−1.39	2.91×10^{-2}
16	MZB1	chr5	−1.37	4.60×10^{-2}
17	GIMAP2	chr7	−1.34	1.42×10^{-3}
18	IL15	chr4	−1.30	2.24×10^{-2}
19	A_33_P3261024	chr6	−1.29	2.94×10^{-2}
20	C10orf10	chr10	−1.29	3.75×10^{-2}
21	IL28A	chr19	−1.29	5.31×10^{-3}
22	C1orf54	chr1	−1.28	3.93×10^{-2}
23	CD74	chr5	−1.28	1.41×10^{-4}
24	GLTSCR2	chr19	−1.24	5.04×10^{-3}
25	ANXA6	chr5	−1.22	1.56×10^{-3}
26	KLHL3	chr5	−1.22	1.57×10^{-2}
27	LOC100240735	chr12	−1.22	4.85×10^{-2}
28	TLR3	chr4	−1.19	8.71×10^{-3}
29	GLI4	chr8	−1.17	3.32×10^{-2}
30	DNHD1	chr11	−1.17	3.14×10^{-2}

Table S5. List of significantly deregulated transcripts in MDS patients with vs. without a SF3B1 mutation (|logFC| > 0.3, FDR < 0.05). logFC—binary logarithm of fold change, FDR—false discovery rate.

No.	Transcript	Chromosome	logFC	FDR
lncRNAs upregulated in patients with mutated SF3B1				
1	RP11-380O24.1	chr3	0.64	3.12×10^{-2}
2	AL592435.1	chr1	0.62	2.13×10^{-2}
3	MIR1302-11	chr19	0.56	1.61×10^{-2}
4	LINC00959	chr10	0.56	1.61×10^{-2}
5	AC093415.2	chr3	0.49	2.70×10^{-3}
6	LINC00705	chr10	0.45	4.43×10^{-2}
7	RP11-692D12.1	chr4	0.44	4.43×10^{-2}
8	RP11-809N15.2	chr6	0.42	1.61×10^{-2}
9	RP11-211C9.1	chr8	0.35	1.61×10^{-2}
10	RP11-446J8.1	chr4	0.35	1.60×10^{-2}
11	USP3-AS1	chr15	0.34	1.09×10^{-2}
12	AC005786.7	chr19	0.31	4.97×10^{-2}
lncRNAs downregulated in patients with mutated SF3B1				
1	RP11-710F7.3	chr4	−0.66	4.71×10^{-2}
2	PCBP1-AS1	chr2	−0.60	4.90×10^{-2}
3	RP11-872J21.3	chr14	−0.58	1.97×10^{-2}
4	RP11-348M17.2	chr5	−0.36	4.97×10^{-2}
5	AL163953.3	chr14	−0.34	3.12×10^{-2}
6	LINC00877	chr3	−0.34	4.71×10^{-2}
PCGs upregulated in patients with mutated SF3B1				
1	AB209400	chr20	2.68	1.66×10^{-3}
2	TCAM1P	chr17	1.86	4.49×10^{-2}
3	ZNF541	chr19	1.34	5.60×10^{-3}
4	CLIC2	chrX	0.79	1.95×10^{-2}
5	KLF11	chr2	0.54	2.53×10^{-2}
6	C15orf40	chr15	0.39	2.39×10^{-2}
PCGs downregulated in patients with mutated SF3B1				
1	ZNF883	chr9	−1.49	1.97×10^{-2}
2	LMO1	chr11	−1.49	1.04×10^{-2}
3	GIPC2	chr1	−1.32	6.35×10^{-3}
4	ARHGAP10	chr4	−0.96	4.86×10^{-2}
5	RTF1	chr15	−0.93	1.82×10^{-3}
6	ABCB7	chrX	−0.88	7.08×10^{-3}
7	RECQL	chr12	−0.81	7.83×10^{-3}
8	ACD	chr16	−0.81	3.03×10^{-4}
9	GAGE1	chrX	−0.57	6.35×10^{-3}
10	EAPP	chr14	−0.48	3.96×10^{-2}
11	NNT	chr5	−0.46	2.39×10^{-2}
12	ATP11C	chrX	−0.44	6.35×10^{-3}
13	LOC100129518	chr6	−0.39	2.67×10^{-2}
14	POLG	chr15	−0.32	4.86×10^{-2}

Table S6. List of significantly deregulated transcripts in MDS patients with vs. without a TET2 mutation (|logFC| > 0.3, FDR < 0.05). logFC—binary logarithm of fold change, FDR—false discovery rate.

**No.**	**Transcript**	**Chromosome**	**logFC**	**FDR**
lncRNAs upregulated in patients with mutated TET2				
1	CTD-2231H16.1	chr5	0.56	4.29×10^{-2}
2	VIPR1-AS1	chr3	0.53	2.78×10^{-2}
3	LINC00518	chr6	0.46	2.78×10^{-2}
4	LINC01193	chr15	0.44	4.20×10^{-2}
5	RP5-1109J22.2	chr1	0.40	1.92×10^{-2}
6	RP11-325N19.3	chr15	0.39	8.53×10^{-3}
7	TBX5-AS1	chr12	0.38	1.92×10^{-2}
8	RP11-104J23.2	chr17	0.30	3.45×10^{-2}
lncRNAs downregulated in patients with mutated TET2				
1	WT1-AS	chr11	−2.70	1.92×10^{-2}
2	EMCN-IT1	chr4	−2.11	1.93×10^{-2}
3	RP11-185E8.1	chr3	−1.56	3.64×10^{-2}
4	RP4-773A18.4	chr1	−0.80	4.29×10^{-2}
5	AC004947.2	chr7	−0.54	1.93×10^{-2}
PCGs upregulated in patients with mutated TET2				
1	MAP2K3	chr17	0.49	4.20×10^{-2}
2	RGS8	chr1	0.43	4.20×10^{-2}
3	BPIFA3	chr20	0.36	2.66×10^{-2}
PCGs downregulated in patients with mutated TET2				
1	PLAC1	chrX	−1.86	2.66×10^{-2}
2	FAM83E	chr19	−0.53	4.20×10^{-2}

Table S7. List of significantly deregulated transcripts in MDS patients with vs. without a TP53 mutation (|logFC| > 0.3, FDR < 0.05). logFC—binary logarithm of fold change, FDR—false discovery rate, ns—nonsignificant transcript.

No.	Transcript	Chromosome	logFC	FDR
lncRNAs upregulated in patients with mutated TP53				
ns	ns	ns	ns	ns
lncRNAs downregulated in patients with mutated TP53				
1	STARD4-AS1	chr5	−1.11	3.83×10^{-2}
2	RP5-1050D4.5	chr17	−0.89	2.03×10^{-2}
3	RP11-53I6.3	chr18	−0.87	2.03×10^{-2}
4	RP11-347I19.8	chr12	−0.81	3.72×10^{-2}
5	RP11-325L7.2	chr5	−0.67	4.47×10^{-2}
6	RP11-57H14.2	chr10	−0.63	3.88×10^{-2}
7	RP11-351M16.3	chr10	−0.60	2.03×10^{-2}
8	RP11-169E6.1	chr16	−0.40	4.11×10^{-2}
PCGs upregulated in patients with mutated TP53				
ns	ns	ns	ns	ns
PCGs downregulated in patients with mutated TP53				
ns	ns	ns	ns	ns

Table S8. List of significantly deregulated transcripts in MDS patients with vs. without a DNMT3A mutation (|logFC| > 0.3, FDR < 0.05). logFC—binary logarithm of fold change, FDR—false discovery rate, ns—nonsignificant transcript.

No.	Transcript	Chromosome	logFC	FDR
lncRNAs upregulated in patients with mutated DNMT3A				
1	RP11-68L1.1	chr3	0.31	4.06E−03
lncRNAs downregulated in patients with mutated DNMT3A				
ns	ns	ns	ns	ns
PCGs upregulated in patients with mutated DNMT3A				
ns	ns	ns	ns	ns
PCGs downregulated in patients with mutated DNMT3A				
ns	ns	ns	ns	ns

Table S9. List of significantly deregulated transcripts in MDS patients with vs. without RUNX1 mutation (|logFC| > 0.3, FDR < 0.05). Of the 67 upregulated and 39 downregulated lncRNAs and the 206 upregulated and 440 downregulated PCGs, only the top 30 transcripts in each category are listed. logFC—binary logarithm of fold change, FDR—false discovery rate.

No.	Transcript	Chromosome	logFC	FDR
lncRNAs upregulated in patients with mutated RUNX1				
1	AC068057.2	chr2	2.65	8.76×10^{-3}
2	C9orf106	chr9	2.00	2.08×10^{-2}
3	RP11-66B24.1	chr15	1.62	4.11×10^{-3}
4	LINC01071	chr13	1.50	3.96×10^{-3}
5	FAM225B	chr9	1.36	3.07×10^{-2}
6	AJ271736.10	chrX	1.22	3.38×10^{-3}
7	WASIR2	chr16	1.13	2.22×10^{-3}
8	RBPMS-AS1	chr8	1.08	4.49×10^{-2}
9	RP11-433M22.1	chr17	1.06	2.96×10^{-2}
10	RP11-490M8.1	chr2	1.01	1.62×10^{-2}
11	HCG9	chr6	1.00	1.89×10^{-2}
12	RP11-750H9.5	chr11	0.92	4.95×10^{-5}
13	RP11-374M1.4	chr9	0.88	3.78×10^{-2}
14	RP11-834C11.3	chr12	0.88	3.63×10^{-2}
15	RP11-783L4.1	chr14	0.87	2.48×10^{-2}
16	LINC01257	chr12	0.81	3.28×10^{-2}
17	RP11-626G11.3	chr16	0.85	4.11×10^{-3}
18	RP11-527L4.5	chr17	0.83	5.14×10^{-3}
19	RP1-309F20.3	chr20	0.81	3.59×10^{-2}
20	RP11-65J3.15	chr9	0.81	4.22×10^{-2}
21	PAN3-AS1	chr13	0.81	4.27×10^{-2}
22	RP5-1024C24.1	chr11	0.81	1.49×10^{-2}
23	PRKAG2-AS1	chr7	0.81	6.36×10^{-3}
24	RP11-650P15.1	chr18	0.81	3.90×10^{-2}
25	RP1-122P22.2	chr20	0.81	4.27×10^{-2}
26	HCP5	chr6	0.78	2.77×10^{-2}
27	LA16c-321D4.2	chr16	0.75	4.42×10^{-2}
28	FAM95B1	chr9	0.75	2.48×10^{-2}
29	GAS5	chr1	0.73	3.64×10^{-3}
30	LINC00954	chr2	0.70	1.90×10^{-2}
lncRNAs downregulated in patients with mutated RUNX1				
1	AL928768.3	chr14	−5.94	4.49×10^{-2}
2	LINC01013	chr6	−4.20	1.13×10^{-3}
3	TCL6	chr14	−4.11	4.11×10^{-3}
4	RP11-542K23.7	chr9	−4.02	4.11×10^{-3}
5	LEF1-AS1	chr4	−2.85	3.39×10^{-3}
6	RP11-222A5.1	chr1	−2.12	1.84×10^{-2}
7	RP11-161M6.2	chr16	−2.09	2.37×10^{-2}
8	RP11-161M6.2	chr16	−2.09	4.19×10^{-2}
9	RP11-175K6.1	chr5	−1.94	1.20×10^{-2}
10	RP11-558A11.3	chr16	−1.86	3.60×10^{-2}
11	RP11-56F10.3	chr9	−1.78	1.13×10^{-3}
12	LINC01218	chr4	−1.54	4.84×10^{-2}
13	PCED1B-AS1	chr12	−1.37	4.49×10^{-2}
14	RP11-78B10.2	chr1	−1.25	4.11×10^{-3}
15	RP11-394O9.1	chr9	−1.25	4.87×10^{-2}

16	RP11-435B5.4	chr1	−1.20	4.10×10^{-2}
17	RP11-384O8.1	chr2	−0.90	2.46×10^{-2}
18	AC005307.1	chr19	−0.89	4.68×10^{-2}
19	GS1-421I3.2	chrX	−0.83	2.66×10^{-2}
20	LINC00226	chr14	−0.82	3.10×10^{-2}
21	RP11-83N9.5	chr9	−0.81	2.14×10^{-2}
22	RP11-417J8.3	chr1	−0.72	4.05×10^{-2}
23	RP11-664D1.1	chr12	−0.70	4.15×10^{-2}
24	RP11-584P21.2	chr4	−0.69	4.99×10^{-2}
25	AC019221.4	chr2	−0.69	4.05×10^{-2}
26	AC007381.3	chr2	−0.63	2.60×10^{-2}
27	CTC-444N24.13	chr19	−0.60	2.91×10^{-2}
28	RP11-527H14.4	chr18	−0.59	1.89×10^{-2}
29	CTD-2384A14.1	chr14	−0.52	4.25×10^{-2}
30	RP11-619L12.3	chr5	−0.47	3.10×10^{-2}
PCGs upregulated in patients with mutated RUNX1				
1	SCARA3	chr8	2.44	4.41×10^{-2}
2	BAI1	chr8	1.87	4.64×10^{-2}
3	ANKRD65	chr1	1.85	4.97×10^{-2}
4	HTRA3	chr4	1.77	2.19×10^{-2}
5	PRDM16	chr1	1.56	1.07×10^{-2}
6	MAMDC2	chr9	1.53	3.49×10^{-2}
7	KBTBD12	chr3	1.37	2.77×10^{-2}
8	GIMAP2	chr7	1.22	2.77×10^{-2}
9	CPNE7	chr16	1.20	2.49×10^{-2}
10	LRP6	chr12	1.17	3.39×10^{-5}
11	GPR162	chr12	1.17	4.97×10^{-2}
12	LOC100134167	chr9	1.17	1.26×10^{-2}
13	AK130024	chr17	1.15	4.44×10^{-6}
14	LINC00256B	chr9	1.10	4.56×10^{-2}
15	CCDC149	chr4	1.10	2.05×10^{-2}
16	PRKCA	chr17	1.09	8.18×10^{-3}
17	LRRD1	chr7	1.08	4.18×10^{-2}
18	LOC100288911	chr2	1.06	7.15×10^{-3}
19	ANPEP	chr15	1.05	4.57×10^{-2}
20	HLA-DQB1	chr6	1.05	3.69×10^{-2}
21	UBA7	chr3	1.04	2.79×10^{-2}
22	HLA-DOA	chr6	1.04	8.18×10^{-3}
23	ISG20	chr15	1.04	1.36×10^{-2}
24	GNPDA1	chr5	1.03	2.31×10^{-2}
25	HSBP1L1	chr18	0.99	4.12×10^{-4}
26	GALNT14	chr2	0.98	1.88×10^{-2}
27	KCNE3	chr11	0.97	4.54×10^{-3}
28	BTG2	chr1	0.95	1.69×10^{-2}
29	FBXO15	chr18	0.93	4.16×10^{-2}
30	C1orf151-NBL1	chr1	0.93	7.97×10^{-3}
PCGs downregulated in patients with mutated RUNX1				
1	POU4F1	chr13	−5.83	2.27×10^{-2}
2	LEF1	chr4	−5.03	2.75×10^{-6}
3	NPY	chr7	−4.73	4.44×10^{-6}
4	IGKV116	chr2	−4.69	2.77×10^{-2}
5	IGKV1D-43	chr2	−4.39	1.67×10^{-2}

6	IGLV1-47	chr22	−4.05	2.19×10^{-2}
7	IGKV1D-8	chr2	−3.98	2.23×10^{-2}
8	RAG1	chr11	−3.96	1.02×10^{-4}
9	AB363267	chr2	−3.91	2.55×10^{-3}
10	IGLV1-44	chr22	−3.87	3.42×10^{-2}
11	IGHV1-18	chr14	−3.78	2.75×10^{-6}
12	NP113779	chr2	−3.68	2.97×10^{-2}
13	LOC100653210	chr2	−3.42	7.87×10^{-3}
14	AF194718	chr22	−3.41	2.04×10^{-2}
15	IGLV3-10	chr22	−3.38	1.18×10^{-2}
16	IRX1	chr5	−3.28	2.96×10^{-2}
17	DUSP26	chr8	−3.25	1.55×10^{-2}
18	IGLV3-9	chr22	−3.19	2.42×10^{-2}
19	COL6A5	chr3	−3.18	9.11×10^{-8}
20	IGLV3-25	chr22	−3.17	1.75×10^{-2}
21	IGKV1D-16	chr2	−3.17	6.81×10^{-3}
22	IGHV1-2	chr14	−3.03	8.80×10^{-6}
23	IGKV1D-27	chr22	−2.98	1.36×10^{-2}
24	IGKV1D-8	chr2	−2.88	5.22×10^{-3}
25	MECOM	chr3	−2.79	2.90×10^{-2}
26	TGM2	chr20	−2.79	4.03×10^{-2}
27	SH2D4B	chr10	−2.78	1.76×10^{-2}
28	IFI27	chr14	−2.77	4.51×10^{-2}
29	ECEL1P2	chr2	−2.75	7.51×10^{-4}
30	NPTX2	chr7	−2.74	1.71×10^{-2}

Table S10. List of significantly deregulated transcripts in patients with long vs. short survival (|logFC| > 1, FDR < 0.05). The cut-off for patient stratification was 18 months from the time of sample collection. logFC—binary logarithm of fold change, FDR—false discovery rate.

No.	Transcript	Chromosome	logFC	FDR
lncRNAs upregulated in patients with short survival				
1	H19	chr11	3.13	1.10×10^{-2}
2	WT1-AS	chr11	1.21	1.03×10^{-2}
3	AC093818.1	chr2	1.20	1.25×10^{-2}
4	ITGA6-AS1	chr2	1.06	3.12×10^{-2}
5	LBX2-AS1	chr2	1.02	3.12×10^{-2}
lncRNAs downregulated in patients with short survival				
1	RP11-121P10.1	chr6	−2.48	3.12×10^{-2}
2	LINC01122	chr2	−1.48	1.25×10^{-2}
3	RP11-120K24.3	chr13	−1.35	2.66×10^{-2}
PCGs upregulated in patients with short survival				
1	PDE3B	chr11	1.61	2.96×10^{-3}
2	GPR124	chr8	1.56	4.41×10^{-2}
3	HIC1	chr17	1.45	2.84×10^{-3}
4	CD97	chr19	1.05	2.84×10^{-3}
5	FLJ90757	chr17	1.01	4.86×10^{-2}
PCGs downregulated in patients with short survival				
1	ECEL1P2	chr2	−2.45	3.88×10^{-4}
2	TCEAL2	chrX	−2.27	3.87×10^{-4}
3	ST6GAL2	chr2	−1.90	1.15×10^{-2}
4	SLC1A6	chr19	−1.86	3.95×10^{-3}
5	PDZK1IP1	chr1	−1.70	5.69×10^{-3}
6	SH3GL3	chr15	−1.69	2.81×10^{-2}
7	TM7SF4	chr8	−1.66	4.41×10^{-2}
8	HLF	chr17	−1.64	8.92×10^{-3}
9	CDH7	chr18	−1.58	5.56×10^{-3}
10	CLCN4	chrX	−1.57	4.41×10^{-2}
11	NFIB	chr9	−1.52	6.39×10^{-3}
12	CXorf57	chrX	−1.45	8.24×10^{-3}
13	STAC	chr3	−1.44	5.69×10^{-3}
14	C3orf14	chr3	−1.43	8.24×10^{-3}
15	TMSB15A	chrX	−1.39	1.33×10^{-2}
16	PRKG2	chr4	−1.37	2.18×10^{-2}
17	AVP	chr20	−1.34	4.41×10^{-2}
18	THC2656240	chrX	−1.34	1.13×10^{-3}
19	JAM2	chr21	−1.32	5.69×10^{-3}
20	MCF2L-AS1	chr13	−1.30	8.24×10^{-3}
21	PCDH9	chr13	−1.21	4.86×10^{-2}
22	RP11-551L14.1	chr12	−1.12	2.11×10^{-2}
23	C7orf58	chr7	−1.02	3.12×10^{-2}
24	VWCE	chr11	−1.00	4.86×10^{-2}

Table S11. List of significantly deregulated transcripts in MDS patients with lower- vs. higher-risk IPSS-R (|logFC| > 1, FDR < 0.05). Of the 67 significantly downregulated PCGs, only the top 30 transcripts are listed. logFC—binary logarithm of fold change, FDR—false discovery rate.

No.	Transcript	Chromosome	logFC	FDR
lncRNAs upregulated in higher-risk MDS patients				
1	RP11-897M7.1	chr12	1.27	4.31×10^{-2}
2	LINC00539	chr13	1.02	4.03×10^{-2}
lncRNAs downregulated in higher-risk MDS patients				
1	TCL6	chr14	−4.48	4.73×10^{-2}
2	LINC01013	chr6	−4.33	2.46×10^{-2}
3	LEF1-AS1	chr4	−1.96	4.29×10^{-2}
4	CTC-436K13.2	chr5	−1.95	4.83×10^{-2}
5	RP11-474N8.5	chr12	−1.88	4.29×10^{-2}
6	AC096579.7	chr2	−1.84	2.46×10^{-2}
7	RP11-879F14.2	chr18	−1.55	4.73×10^{-2}
8	RP11-69I8.3	chr6	−1.35	4.04×10^{-2}
9	LINC01037	chr1	−1.34	4.91×10^{-2}
10	RP11-401P9.5	chr16	−1.26	4.73×10^{-2}
11	AC147651.3	chr7	−1.20	4.73×10^{-2}
12	RP11-71G12.1	chr1	−1.08	1.47×10^{-2}
13	RP3-523C21.2	chr6	−1.05	4.73×10^{-2}
14	CTA-250D10.23	chr22	−1.02	4.29×10^{-2}
PCGs upregulated in higher-risk MDS patients				
1	BAI1	chr8	2.56	7.51×10^{-3}
2	ARC	chr8	2.55	1.32×10^{-2}
3	PTH2R	chr2	1.83	2.17×10^{-2}
4	MAMDC2	chr9	1.77	4.23×10^{-2}
5	LOXL4	chr10	1.67	4.40×10^{-2}
6	NPM2	chr8	1.36	1.58×10^{-2}
7	A_33_P3387493	chrX	1.30	4.41×10^{-2}
8	A_24_P247454	chr2	1.29	3.60×10^{-2}
9	KRT18	chr4	1.23	2.77×10^{-2}
10	A_24_P230057	chrX	1.22	2.30×10^{-2}
11	A_24_P401601	chr19	1.20	9.47×10^{-3}
12	KRT18P55	chr17	1.17	2.29×10^{-2}
13	A_24_P358131	chr2	1.15	4.34×10^{-3}
14	A_33_P3240295	chr2	1.06	8.75×10^{-3}
15	FLJ90757	chr17	1.02	1.17×10^{-2}
PCGs downregulated in higher-risk MDS patients				
1	RAG1	chr11	−3.91	1.49×10^{-2}
2	NPY	chr7	−3.71	1.34×10^{-2}
3	DUSP26	chr8	−3.39	1.20×10^{-2}
4	IGHV1-18	chr14	−3.25	2.77×10^{-2}
5	IGHV1-2	chr14	−3.01	3.86×10^{-2}
6	STAB2	chr12	−2.88	2.31×10^{-2}
7	CD24	chrY	−2.79	2.18×10^{-2}
8	LEF1	chr4	−2.73	7.96×10^{-3}
9	SPANXB2	chrX	−2.59	2.42×10^{-2}
10	AKAP12	chr6	−2.50	1.34×10^{-2}
11	CHST15	chr10	−2.46	8.75×10^{-3}
12	RBP7	chr1	−2.42	2.62×10^{-2}
13	SLAMF7	chr1	−2.40	2.36×10^{-2}

14	MME	chr3	−2.37	4.44×10^{-2}
15	LOC283454	chr12	−2.35	2.95×10^{-2}
16	TFF3	chr21	−2.20	2.30×10^{-2}
17	CTTN	chr11	−2.19	3.97×10^{-3}
18	IGJ	chr4	−2.15	2.30×10^{-2}
19	BTNL9	chr5	−2.12	1.41×10^{-2}
20	NR2F2	chr15	−2.10	4.87×10^{-2}
21	THC2750292	chr6	−2.06	2.25×10^{-2}
22	CLEC4G	chr19	−2.05	4.41×10^{-2}
23	NGFR	chr17	−1.95	7.51×10^{-3}
24	SLC35D3	chr6	−1.87	2.30×10^{-2}
25	CTGF	chr6	−1.86	1.17×10^{-2}
26	NUAK1	chr12	−1.86	2.77×10^{-2}
27	PDE5A	chr4	−1.86	8.75×10^{-3}
28	IL28RA	chr1	−1.76	4.42×10^{-3}
29	CLCN4	chrX	−1.73	2.12×10^{-2}
30	CLDN5	chr22	−1.73	2.77×10^{-2}

Table S12. Correlations of lncRNA expression with clinical variables of MDS patients. Spearman correlation coefficients (r) are shown for each pair of variables. (D) Discovery cohort, (T) testing cohort.

Correlation Coefficient (*r*)	H19 Level	WT1-AS Level	LEF1-AS1 Level	TCL6 Level	TP53 Mutation
Age	(D) −0.142<br>(T) 0.073	(D) −0.118<br>(T) 0.022	(D) −0.134<br>(T) −0.288 *	(D) 0.060<br>(T) 0.1415	(D) 0.094<br>(T) n.a.
Marrow blast count	(D) 0.149<br>(T) 0.118	(D) 0.269 *<br>(T) 0.284 **	(D) −0.383 **<br>(T) −0.297 **	(D) −0.214<br>(T) −0.249 **	(D) 0.238<br>(T) n.a.
Hemoglobin level	(D) 0.201<br>(T) −0.174	(D) 0.006<br>(T) −0.016	(D) 0.105<br>(T) 0.149	(D) 0.130<br>(T) −0.006	(D) −0.316 *<br>(T) n.a.
Neutrophil count	(D) −0.022<br>(T) −0.023	(D) −0.176<br>(T) −0.309 **	(D) −0.087<br>(T) 0.188	(D) −0.084<br>(T) −0.098	(D) −0.234<br>(T) n.a.
Platelet count	(D) −0.163<br>(T) −0.254 *	(D) −0.180<br>(T) −0.191	(D) 0.123<br>(T) 0.176	(D) 0.081<br>(T) −0.209	(D) −0.214<br>(T) n.a.
H19 level		(D) 0.141<br>(T) 0.187	(D) −0.102<br>(T) −0.045	(D) −0.054<br>(T) −0.112	(D) −0.028<br>(T) n.a.
WT1-AS level			(D) −0.034<br>(T) −0.322 **	(D) −0.058<br>(T) −0.078	(D) 0.445 **<br>(T) n.a.
LEF1-AS1 level				(D) 0.851 ***<br>(T) 0.580 ***	(D) −0.130<br>(T) n.a.
TCL6 level					(D) −0.127<br>(T) n.a.

n.a.—not assessed. Significant correlations are marked (* $p < 0.05$, ** $p < 0.01$, *** $p < 0.001$).

References

1. Arber, D.A.; Orazi, A.; Hasserjian, R.; Thiele, J.; Borowitz, M.J.; Le Beau, M.M.; Bloomfield, C.D.; Cazzola, M.; Vardiman, J.W. The 2016 revision to the World Health Organization classification of myeloid neoplasms and acute leukemia. *Blood* **2016**, *127*, 2391–2405, doi:10.1182/blood-2016-03-643544.
2. Greenberg, P.L.; Tuechler, H.; Schanz, J.; Sanz, G.; Garcia-Manero, G.; Solé, F.; Bennett, J.M.; Bowen, D.; Fenaux, P.; Dreyfus, F.; et al. Revised International Prognostic Scoring System for Myelodysplastic Syndromes. *Blood* **2012**, *120*, 2454–2465, doi:10.1182/blood-2012-03-420489.
3. Derrien, T.; Johnson, R.; Bussotti, G.; Tanzer, A.; Djebali, S.; Tilgner, H.; Guernec, G.; Martín, D.; Merkel, A.; Knowles, D.G.; et al. The GENCODE v7 catalog of human long noncoding RNAs: Analysis of their gene structure, evolution, and expression. *Genome Res.* **2012**, *22*, 1775–1789, doi:10.1101/gr.132159.111.
4. Saeed, A.I.; Bhagabati, N.K.; Braisted, J.C.; Liang, W.; Sharov, V.; Howe, E.A.; Li, J.; Thiagarajan, M.; White, J.A.; Quackenbush, J. [9] TM4 Microarray Software Suite. *Methods Enzym.* **2006**, *411*, 134–193, doi:10.1016/s0076-6879(06)11009-5.
5. Xie, F.; Xiao, P.; Chen, N.; Xu, L.; Zhang, B. miRDeepFinder: a miRNA analysis tool for deep sequencing of plant small RNAs. *Plant Mol. Biol.* **2012**, *80*, 75–84, doi:10.1007/s11103-012-9885-2.
6. Livak, K.J.; Schmittgen, T.D. Analysis of relative gene expression data using real-time quantitative PCR and the 2(-Delta Delta C(T)) Method. *Methods* **2001**, *25*, 402–408.
7. Subramanian, A.; Tamayo, P.; Mootha, V.K.; Mukherjee, S.; Ebert, B.L.; Gillette, M.A.; Paulovich, A.; Pomeroy, S.L.; Golub, T.R.; Lander, E.S.; et al. Gene set enrichment analysis: A knowledge-based approach for interpreting genome-wide expression profiles. *Proc. Natl. Acad. Sci. USA* **2005**, *102*, 15545–15550, doi:10.1073/pnas.0506580102.
8. Liu, K.; Beck, D.; Thoms, J.A.I.; Liu, L.; Zhao, W.; Pimanda, J.E.; Zhou, X. Annotating function to differentially expressed LincRNAs in myelodysplastic syndrome using a network-based method. *Bioinform.* **2017**, *33*, 2622–2630, doi:10.1093/bioinformatics/btx280.
9. Brunet, J.-P.; Tamayo, P.; Golub, T.R.; Mesirov, J.P. Metagenes and molecular pattern discovery using matrix factorization. *Proc. Natl. Acad. Sci. USA* **2004**, *101*, 4164–4169, doi:10.1073/pnas.0308531101.
10. Wierenga, A.T.J.; Vellenga, E.; Schuringa, J.J. Maximal STAT5-Induced Proliferation and Self-Renewal at Intermediate STAT5 Activity Levels. *Mol. Cell. Biol.* **2008**, *28*, 6668–6680, doi:10.1128/mcb.01025-08.
11. Jaatinen, T.; Hemmoranta, H.; Hautaniemi, S.; Niemi, J.; Nicorici, D.; Laine, J.; Yli-Harja, O.; Partanen, J. Global Gene Expression Profile of Human Cord Blood-Derived CD133+Cells. *Stem Cells* **2006**, *24*, 631–641, doi:10.1634/stemcells.2005-0185.
12. Ross, M.E.; Mahfouz, R.; Onciu, M.; Liu, H.-C.; Zhou, X.; Song, G.; Shurtleff, S.A.; Pounds, S.; Cheng, C.; Ma, J.; et al. Gene expression profiling of pediatric acute myelogenous leukemia. *Blood* **2004**, *104*, 3679–3687, doi:10.1182/blood-2004-03-1154.
13. Eppert, K.; Takenaka, K.; Lechman, E.R.; Waldron, L.; Nilsson, B.; Van Galen, P.; Metzeler, K.H.; Poeppl, A.; Ling, V.; Beyene, J.; et al. Stem cell gene expression programs influence clinical outcome in human leukemia. *Nat. Med.* **2011**, *17*, 1086–1093, doi:10.1038/nm.2415.
14. Graham, S.M.; Vass, J.K.; Holyoake, T.L.; Graham, G.J. Transcriptional Analysis of Quiescent and Proliferating CD34+ Human Hemopoietic Cells from Normal and Chronic Myeloid Leukemia Sources. *Stem Cells* **2007**, *25*, 3111–3120, doi:10.1634/stemcells.2007-0250.
15. Lim, E.; Wu, D.; Pal, B.; Bouras, T.; Asselin-Labat, M.-L.; Vailllant, F.; Yagita, H.; Lindeman, G.J.; Smyth, G.K.; Visvader, J.E. Transcriptome analyses of mouse and human mammary cell subpopulations reveal multiple conserved genes and pathways. *Breast Cancer Res.* **2010**, *12*, R21, doi:10.1186/bcr2560.
16. Massarweh, S.; Osborne, C.K.; Creighton, C.J.; Qin, L.; Tsimelzon, A.; Huang, S.; Weiss, H.; Rimawi, M.; Schiff, R. Tamoxifen Resistance in Breast Tumors Is Driven by Growth Factor Receptor Signaling with Repression of Classic Estrogen Receptor Genomic Function. *Cancer Res.* **2008**, *68*, 826–833, doi:10.1158/0008-5472.can-07-2707.
17. Wang, Z.; Iwasaki, M.; Ficara, F.; Lin, C.; Matheny, C.; Wong, S.H.K.; Smith, K.S.; Cleary, M.L. GSK-3 Promotes Conditional Association of CREB and Its Coactivators with MEIS1 to Facilitate HOX-Mediated Transcription and Oncogenesis. *Cancer Cell* **2010**, *17*, 597–608, doi:10.1016/j.ccr.2010.04.024.

18. Haddad, R. Molecular characterization of early human T/NK and B-lymphoid progenitor cells in umbilical cord blood. *Blood* **2004**, *104*, 3918–3926, doi:10.1182/blood-2004-05-1845.
19. Yoshimura, K.; Aoki, H.; Ikeda, Y.; Fujii, K.; Akiyama, N.; Furutani, A.; Hoshii, Y.; Tanaka, N.; Ricci, R.; Ishihara, T.; et al. Regression of abdominal aortic aneurysm by inhibition of c-Jun N-terminal kinase. *Nat. Med.* **2005**, *11*, 1330–1338, doi:10.1038/nm1335.
20. Martens, J.H.A.; Brinkman, A.B.; Simmer, F.; Francoijs, K.; Nebbioso, A.; Ferrara, F.; Altucci, L.; Stunnenberg, H.G. PML-RARalpha/RXR Alters the Epigenetic Landscape in Acute Promyelocytic Leukemia. *Cancer Cell* 2010, *17*, 173–185.
21. Heller, G.; Schmidt-Erfurth, U.; Ziegler, B.; Hölzer, S.; Müllauer, L.; Bilban, M.; Zielinski, C.; Drach, J.; Zöchbauer-Müller, S. Genome-Wide Transcriptional Response to 5-Aza-2'-Deoxycytidine and Trichostatin A in Multiple Myeloma Cells. *Cancer Res.* **2008**, *68*, 44–54, doi:10.1158/0008-5472.can-07-2531.
22. Mikkelsen, T.S.; Ku, M.; Jaffe, D.B.; Issac, B.; Lieberman, E.; Giannoukos, G.; Alvarez, P.; Brockman, W.; Kim, T.-K.; Koche, R.P.; et al. Genome-wide maps of chromatin state in pluripotent and lineage-committed cells. *Nat.* **2007**, *448*, 553–560, doi:10.1038/nature06008.
23. Meissner, A.; Mikkelsen, T.S.; Gu, H.; Wernig, M.; Hanna, J.; Sivachenko, A.; Zhang, X.; Bernstein, B.E.; Nusbaum, C.; Jaffe, D.B.; et al. Genome-scale DNA methylation maps of pluripotent and differentiated cells. *Nature* **2008**, *454*, 766–770, doi:10.1038/nature07107.
24. Diaz-Blanco, E.; Bruns, I.; Neumann, F.; Fischer, J.C.; Graef, T.; Rosskopf, M.; Brors, B.; Pechtel, S.; Bork, S.; Koch, A.; et al. Molecular signature of CD34+ hematopoietic stem and progenitor cells of patients with CML in chronic phase. *Leukemia* **2007**, *21*, 494–504, doi:10.1038/sj.leu.2404549.
25. Piccaluga, P.P.; Agostinelli, C.; Califano, A.; Carbone, A.; Fantoni, L.; Ferrari, S.; Gazzola, A.; Gloghini, A.; Righi, S.; Rossi, M.; et al. Gene Expression Analysis of Angioimmunoblastic Lymphoma Indicates Derivation from T Follicular Helper Cells and Vascular Endothelial Growth Factor Deregulation. *Cancer Res.* **2007**, *67*, 10703–10710, doi:10.1158/0008-5472.can-07-1708.
26. Kumar, A.R.; Hudson, W.A.; Chen, W.; Nishiuchi, R.; Yao, Q.; Kersey, J.H. Hoxa9 influences the phenotype but not the incidence of Mll-AF9 fusion gene leukemia. *Blood* **2004**, *103*, 1823–1828, doi:10.1182/blood-2003-07-2582.

© 2020 by the authors. Licensee MDPI, Basel, Switzerland. This article is an open access article distributed under the terms and conditions of the Creative Commons Attribution (CC BY) license (http://creativecommons.org/licenses/by/4.0/).
